# A meta-QTL analysis highlights genomic hotspots associated with phosphorus use efficiency in rice (*Oryza sativa* L.)

**DOI:** 10.3389/fpls.2023.1226297

**Published:** 2023-08-18

**Authors:** Ian Paul Navea, Phyu Phyu Maung, Shiyi Yang, Jae-Hyuk Han, Wen Jing, Na-Hyun Shin, Wenhua Zhang, Joong Hyoun Chin

**Affiliations:** ^1^ Food Crops Molecular Breeding Laboratory, Department of Integrative Biological Sciences and Industry, Sejong University, Seoul, Republic of Korea; ^2^ Convergence Research Center for Natural Products, Sejong University, Seoul, Republic of Korea; ^3^ College of Life Sciences, National Key Laboratory of Crop Genetics & Germplasm Enhancement and Utilization, Nanjing Agricultural University, Nanjing, China; ^4^ The International Rice Research Institute-Korea Office, National Institute of Crop Science, Rural Development Administration, Iseo-myeon, Republic of Korea

**Keywords:** rice, phosphorus use efficiency, Meta-QTL analysis, Quantitative Trait Loci, candidate genes, superior haplotypes

## Abstract

Phosphorus use efficiency (PUE) is a complex trait, governed by many minor quantitative trait loci (QTLs) with small effects. Advances in molecular marker technology have led to the identification of QTLs underlying PUE. However, their practical use in breeding programs remains challenging due to the unstable effects in different genetic backgrounds and environments, interaction with soil status, and linkage drag. Here, we compiled PUE QTL information from 16 independent studies. A total of 192 QTLs were subjected to meta-QTL (MQTL) analysis and were projected into a high-density SNP consensus map. A total of 60 MQTLs, with significantly reduced number of initial QTLs and confidence intervals (CI), were identified across the rice genome. Candidate gene (CG) mining was carried out for the 38 MQTLs supported by multiple QTLs from at least two independent studies. Genes related to amino and organic acid transport and auxin response were found to be abundant in the MQTLs linked to PUE. CGs were cross validated using a root transcriptome database (RiceXPro) and haplotype analysis. This led to the identification of the eight CGs (*OsARF8, OsSPX-MFS3, OsRING141, OsMIOX, HsfC2b, OsFER2, OsWRKY64*, and *OsYUCCA11*) modulating PUE. Potential donors for superior PUE CG haplotypes were identified through haplotype analysis. The distribution of superior haplotypes varied among subspecies being mostly found in *indica* but were largely scarce in *japonica*. Our study offers an insight on the complex genetic networks that modulate PUE in rice. The MQTLs, CGs, and superior CG haplotypes identified in our study are useful in the combination of beneficial alleles for PUE in rice.

## 1 Introduction

Phosphorus (P) is one of the most important macronutrients in plants and is required in large quantities. Inorganic phosphate (Pi) is a crucial component of phospholipids and plays a significant role in deoxyribonucleic acid (DNA) and ribonucleic acid (RNA) synthesis and other nucleotide-containing molecules. Moreover, P present in adenosine triphosphate (ATP), adenosine diphosphate (ADP), and nicotinamide adenine dinucleotide phosphate (NADPH), provides plant cells with energy required for metabolic and catabolic cellular processes (Heuer et al., 2017). In rice (*Oryza sativa*), P deficiency substantially decreases overall productivity by reducing plant height, tiller number, panicle length (Irfan et al., 2020) and suppresses the root growth (Liu, 2021). Delayed maturity, high sterility, and poor grain quality (Ismail et al., 2007) have also been reported.

Approximately 5.7 billion hectares of land lack plant-available P due to its immobility in soil (Dobermann and Fairhurst, 2000). P tends to get fixed in soils with extreme levels of pH due to the complexation of P by aluminum (Al) or iron (Fe) in acidic conditions and by calcium (Ca) in alkaline soils (Haefele et al., 2014). Unlike N, which has an unlimited source due to its abundance in the atmosphere thanks to the Haber-Bosch process (Lynch, 2011), P fertilizer sources are finite, mainly consisting of phosphate rocks that are estimated to last for only 300~400 years (Shimizu et al., 2004; Van Kauwenbergh, 2010).

Plants have evolved to cope with P-starvation by undergoing physiological changes in their root morphology, such as the promotion of lateral root and root hair growth, and the inhibition of primary root development. Additionally, plants foster symbiosis between their roots and arbuscular mycorrhizal fungi to scavenge Pi from the soil (Péret et al., 2011). The uptake of P in plants is determined by three primary factors: (1) root morphology; (2) P uptake efficiency; and (3) internal P-use efficiency. Among these mechanisms, genetic variations in root morphology are the primary causal factor of P uptake, whereas P uptake efficiency and internal P-use contribute less (Ismail et al., 2007). Consequently, enhancing root morphology through breeding efforts may lead to the development of rice varieties with high phosphorus use efficiency (PUE).

Development of P-efficient rice varieties can be achieved through improved uptake of phosphate from soil (P-acquisition efficiency) (Mori et al., 2016) and improved biomass and/or yield per unit P taken up [internal P-utilization efficiency/P-use efficiency] (PUE) (Rose and Wissuwa, 2012). Developing P-efficient rice genotypes has gained significant attention to breeders. The major PUE quantitative trait locus (QTL), *Pup1*, has been extensively utilized due to its large additive effect. The QTL was mapped from a backcross population derived from a cross between the P-deficiency intolerant Nipponbare and P-deficiency tolerant Kasalath and is located on the long arm of chromosome 12. The use of *Pup1* in molecular marker-assisted backcrossing (MABC) has proven successful (Chin et al., 2011). The *Pup1* QTL harbors the Kasalath-derived *OsPSTOL1* gene, encoding a protein kinase that enhances early-stage root morphology in rice (Gamuyao et al., 2012). Similarly, a minor QTL on the long arm of chromosome 6 (Shimizu et al., 2004) where three of the known rice Pi-responsive regulatory genes *OsERF3*, *OsTHS1* (Wasaki et al., 2003), and *OsPTF1* (Yi et al., 2005) collocate, has been mapped but is yet to be implemented in large-scale breeding programs. In addition, numerous promising genes that are involved in PUE have been identified through overexpression and knockout studies in rice and are now undergoing advanced testing in cereal crops (Heuer et al., 2017). These genes include *AVP1* (Yang et al., 2014), *PHO1* (Khan et al., 2014), *OsPHT1;6* (Ai et al., 2009), and *SPX-MFS* (Wang et al., 2015). Moreover, the next-generation sequencing (NGS) has facilitated the development of high-density rice linkage maps, which have been utilized to identify QTLs associated with various rice traits, including PUE. Several studies have used SNP-based linkage maps to identify PUE QTLs (Koide et al., 2013; Ogawa et al., 2014; Navea et al., 2017; Fu et al., 2019; Ranaivo et al., 2022; Wang et al., 2014).

Despite the progress in ascertaining QTLs and genes underlying PUE, their practical utility in breeding programs remain elusive due to their unstable effects across different genetic backgrounds and environments (Arcade et al., 2004; Daware et al., 2017; Navea et al., 2022) as well as their complex relationship with soil status (Heuer et al., 2017) and linkage drag (Kumar et al., 2020). It is therefore necessary to identify genomic regions conferring robust and stable effects across a wide range of genetic background and environments, as well as QTLs with small CI to increase the efficiency and precision of genomics-assisted breeding (Collard and Mackill, 2008).

Meta-QTL analysis is a powerful tool that can help achieve breeding precision by compiling QTLs identified from various mapping populations used in independent studies. It can also provide target genomic regions with considerably small CI (Goffinet and Gerber, 2000; Khahani et al., 2021). Previous studies successfully identified the meta-QTL’s (MQTL) underlying yield (Khahani et al., 2021; Aloryi et al., 2022), nitrogen-use efficiency (Sandhu et al., 2021), salinity tolerance (Islam et al., 2019), grain zinc content (Joshi et al., 2023), drought (Selamat and Nadarajah, 2021), and grain traits (Selamat and Nadarajah, 2021) in rice. However, to date, MQTLs associated with PUE remain to be explored in rice. The present study aims to fill this gap by identifying promising MQTLs through an extensive literature search on previous PUE QTLs mapped in various independent studies and identify potential rice donors for pyramiding of the beneficial alleles of the candidate genes (CGs) identified within the MQTLs.

## 2 Materials and methods

### 2.1 Compilation of PUE QTLs from various studies

An exhaustive literature search on rice QTLs linked to PUE from studies published between 1998 and 2022 was performed using the publicly available QTL databases, such as PubMed (https://pubmed.ncbi.nlm.nih.gov), Google scholar (https://scholar.google.com), and Gramene QTL database (https://archive.gramene.org). QTLs and traits associated with PUE were defined as traits contributing to biomass and yield (Rose and Wissuwa, 2012) under low P condition, such as shoot dry weight (SDW); absolute value of root traits (RT); root-shoot ratio (RSR); total dry biomass (BM); seed P content (SPC); relative response to P (RRP); internal P translocation (IPT); and yield component (YLD). QTLs identified under low P input were chosen for the meta-QTL (MQTL) anlaysis. Additionally, only QTL mapping studies carried out under field conditions were selected. Sixteen independent studies were used to perform the MQTL analysis. A total of 192 PUE QTLs mapped from 15 non-overlapping bi-parental populations were utilized in the analysis. Information on the type of parental lines, population types, size of the mapping population, type of molecular markers, number of QTLs, and trait types were recorded. Individual QTLs were assessed for QTL names, type of mapping population, trait class, year of study, linkage group, logarithm of odds (LOD) scores, phenotypic variance explained (PVE), peak position (cM), and CI. These attributes were used to generate the “QTL file”. The peak positions were inferred from the midpoint between two flanking markers whenever information on the peak position was lacking. An LOD threshold of 3.0 was set when LOD scores were not supplied. Missing CI values were estimated using the following formulas proposed in a previous study (Darvasi and Seller, 1997; Weller and Soller, 2004):


$$\left(\text{I}\right)\text{CI}=\frac{163}{\text{N x PVE}}\;\left(\text{II}\right)\text{CI}=\frac{530}{\text{N x PVE}}$$

Where N = population size and PVE = phenotypic variance explained. Equation I was used to estimate CI in recombinant inbred lines (RILs) whereas equation II was used for populations derived from F_2_, backcross inbred lines (BILs), and chromosome substitution lines (CSSLs). Missing PVE values were calculated using the following formula (Nagelkerke, 1991):


$$\text{PVE}=1-10\;\left(-\frac{2\text{LOD}}{\text{N}}\right)$$

### 2.2 Construction of consensus map and QTL projection

A high-density consensus map was constructed by integrating SNP markers from the 6K Infinium SNP array (Thomson et al., 2017) and the flanking markers of 192 QTLs. The physical locations of the flanking markers were determined by aligning their sequences to the Nipponbare reference genome (IRGSP v. 1.0) using the Basic Local Alignment Tool (https://www.ncbi.nlm.nih.gov).Then, the closest markers from the Infinium SNP array were used to project QTLs on the consensus map. Markers were arranged according to their physical positions. The consensus map was created by converting the physical position into centimorgan units (cM) using the conversion factor of 1 cM = 250 kb (Raghavan et al., 2017). An individual “map file” for each chromosome was generated, containing information on linkage group, marker name, and the genetic position in centimorgan (cM). The QTL and map files were used as input files to project the consolidated QTLs on the consensus map.

### 2.3 Meta-QTL analysis

After QTL projection, MQTL analysis was performed using Biomercator v4.2.3 software (Arcade et al., 2004; Sosnowski et al., 2012). Two different approaches were applied based on the number of QTLs on each chromosome, namely the Goffinet and Gerber method when the number of QTLs was ≤ 10, and the Veyrieras approach when the number of QTLs was > 10 (Goffinet and Gerber, 2000; Veyrieras et al., 2007). The algorithms and statistical procedures for both methods were previously described in detail (Sosnowski et al., 2012). The “true” number of MQTLs per chromosome was defined from the model with the lowest Akaike information criterion (AIC) value, which was selected as it contained the least amount of information loss (Akaike, 1987). The PVE value for each MQTL were estimated using that of its “initial QTL” members. The best model, along with its corresponding AIC values, MQTL peak positions, 95% CI, and physical positions, are presented in (**Table 1**). Additionally, only MQTLs with an average PVE value of ≥ 5% and supported by QTLs identified in at least two independent studies were selected for further analysis. The QTLs identified under P-deficient or P-non-supplied conditions were categorized into the following traits: 1) shoot dry weight (SDW); 2) absolute values of root traits (RT); 3) root-shoot ratio (RSR); 4) total dry biomass (BM); 5) seed P content (SPC); 6) relative response to P (RRP); 7) internal P translocation (IPT); and 8) yield components (YLD).

**Table 1:** QTL studies used in the QTL meta-analysis for PUE in rice.

Parents (Subspecies or species)	Population type^a^	Population size	Type of marker^b^	Number of marker	Number of QTLs identified	Traits^c^	LOD scores/ PVE^d^ (%)	Country of experiment	Year	Reference
Zhonghui9308 x Xieyou9308 (*indica x indica*)	CSSL	75	SSR + InDel	120	7	SDW, RT, SDW, BM.	2.00~3.32 10.82~18.46	China	2018	Anis et al., 2018
ZYQ8 x JX17 (*indica x japonica*)	DH	127	RFLP	444	6	RRP, RT, RSR	2.03~6.79 8.8~25.2	China	2000	Ming et al., 2000
GH128 x W6827 (*indica x japonica*)	F_2_	262	SNP	25,117	21	BM, RRP, YLD,SPC, IPT	2.53~9.50 1.56~8.19	China	2019	Fu et al., 2019
IR20 x IR55178 (*indica x indica*)	RIL	284	AFLP + RFLP	178	6	RRP	3.40~9.10 13~21	China	2001	Hu et al., 2001
Wazuhophek x Samba Mahsuri (*indica x indica*)	RIL	330	SSR	78	15	SDW, YLD, RT, BM, RSR, SPC	5.29~5.84 2.25~21.84	India	2021	Kale et al., 2021
Nerica10 x Hitomebore (*indica x japonica*)	F_2:3_	91	SNP + SSR	128	15	SDW, YLD, BM, IPT	2.60~4.90 12~23.7	Japan	2013	Koide et al., 2013
Mizukagami x OHA15 (*japonica x indica*)	CSSLs + F_2_	35 + 176	SSR	9 (Chr. 6 only)	3	YLD, RT, SDW	2.20~12.70 5.5~27.5	Japan	2022	Kokaji et al., 2022
Dasanbyeo x TR22183 (*indica x japonica*)	RIL	172	SNP	236	30	YLD	3.15~12.76 8.9~22.52	Philippines/Korea	2017	Navea et al., 2017
IR20 x IR55178 (*indica x indica*)	RIL	285	AFLP	217	10	SDW, BM, RRP	2.42~16.98 6.8~19.5	Philippines	1998	Ni et al., 1998
Curinga x IRGC105491 (*O. sativa x O. rufipogon*)	CSSL	48	SNP	238	3	RT	3.0 1.25	Colombia	2014	Ogawa et al., 2014
IRAT109 x Yuefa (*indica x japonica*)	DH	116	RFLP+SSR	165	17	YLD	3.02~5.13 2.65~20.78	China	2008	Ping et al., 2008
DJ123 x Nerica4 (*aus x indica*)	BIL	201	SNP	1578	10	RT, SDW, BM	4.46~7.65 1.5~19.2	Japan and Madagascar	2022	Ranaivo et al., 2022
Gimbozu x Kasalath (*japonica x indica*)	F_2:3_	82	SSR	97	11	SDW, BM, RSR, RRP	3.50~6.46 9.1~24.6	Japan	2004	Shimizu et al., 2004
Zhenshan 97 x Minghui 63 (*indica x indica*)	RIL	113	SNP	1,619	36	BM, YLD, PUP, SPC	3.0 1.4~15.8	China	2014	Wang et al., 2019
Nipponbare x Kasalath (*japonica x indica*)	BIL	98	RFLP	245	13	PUP, SDW, YLD	2.82~10.74 5.8~30	Japan	1998	Wissuwa et al., 1998
Shuhui527 x Yetuozai (*japonica x indica*)	BIL	60	SSR	96	20	RT, RSR, YLD	3.78~6.81 3.6~15.9	China	2015	Zhang et al., 2014

**a CSSL**, chromosome substitution lines; **DH**, doubled haploid lines; **RIL**, recombinant inbred lines; **F_2,3_,** F_2_-derived F_3_ lines; **BIL**, backcross inbred lines.

**b SSR**, single sequence repeats; **RFLP**, restriction fragment length polymorphism; **AFLP**, amplified fragment length polymorphism; **SNP**, single nucleotide polymorphism.

^**c**^ **SDW**, shoot dry weight; **RT**, absolute value of root traits; **RSR,** root-shoot ratio; **BM**, total dry biomass; **SPC**, seed P content; **RRP**, relative response to P; **IPT**, internal P translocation; **YLD**, yield component.

^**d**^ **LOD**,logarithm of odds; **PVE**, phenotypic variation explained.

### 2.4 Identification of CGs underlying PUE in rice

After identifying MQTLs with an average PVE value of ≥ 5% and support from at least two independent studies using the model with the lowest AIC value, we aimed to mine CGs associated with PUE in rice. To this end, we utilized the physical position of markers flanking the MQTLs as query terms in the Rice Annotation Project Database (IRGSP v1.0 and MSU 7) to batch download functionally annotated gene models within the MQTLs. All genes within the MQTLs were initially considered as CGs, which were further filtered based on gene ontology (GO) terms and/or keywords related to PUE such as P homeostasis, phosphate, inorganic phosphate (Pi) transporter, crown root, root hairs, phosphorus translocation, phosphorus uptake, abiotic stress, and/or secondary traits associated with PUE, as described in a review article by Heuer et al. (2017).

To identify P-responsive genes, we conducted *in silico* analysis on the CGs identified in the previous step. We used a microarray dataset (RXP_5002, available at https://ricexpro.dna.affrc.go.jp) containing root gene expression data of 7-day-old seedlings grown under P-deficient and control conditions. Details of the plant growth conditions and treatments can be found on the RiceXPro website (https://ricexpro.dna.affrc.go.jp/RXP_5002/details-of-methods.html). Briefly, 7-day-old Nipponbare seedlings were exposed to P-non-supplied and control (P-supplied) conditions. Root samples were collected at 6- and 24-hour (h) post-treatment for RNA extraction. The RNA was labeled with Cy3 and subjected to hybridization using the Agilent one-color microarray analysis system. The resulting gene expression profile was presented in terms of raw signal intensity. We carried out gene ontology (GO) enrichment analysis using the Shiny GO 0.76 database (Ge et al., 2020) to determine the most enriched biological pathways in the P-responsive genes with at least 1.5-fold change in expression (P-non-supplied/control condition). We used all the unfiltered genes within the MQTLs as the background against the P-responsive genes in the GO enrichment analysis. In the next step, we further filtered the CGs to those that were responsive to P at both 6- and 24-h post-treatment, with a consistent direction of regulation at both time points, and at least a 2-fold change in expression levels at 24-h post-treatment (P-non-supplied relative to control).

### 2.5 Statistical analysis

Statistical analysis was performed using the two-tailed t-test to determine the significance of differences in the root gene expression levels at different P application at P<0.10, P<0.05, and P<0.01 using Minitab release v.14.

### 2.6 Identification of potential donors for the pyramiding of beneficial CG alleles

Haplotype analysis carried out for the PUE CGs with significant responses to P treatment was performed using the SNP Seek database’s built-in tool (Mansueto et al., 2016) in the 3K Rice Genome Project (RGP) database. The database included all rice sub-populations with Nipponbare as the reference genome. PUE CGs with non-synonymous SNPs, at least a 2-fold change in gene expression under P-deficient vs. control conditions, and with at least two haplotypes were utilized in the analysis. The number of haplotypes were determined using the default parameters with the Calinski criterion for determining the optimal number of groups (Caliñski and Harabasz, 1974). Haplotypes with fewer than three genotypes were excluded. Genotypes with more than 20% missing SNPs or heterozygous loci were filtered out from the data set. Haplotypes identical to those of the beneficial allele donors (whenever available in the 3K RGP dataset) in the QTL studies used in the MQTL analysis were regarded as superior haplotypes. The abundance of superior haplotypes across the subpopulations was calculated, and rice accessions with superior haplotypes in *indica*, *japonica*, and *aus* backgrounds from the 3K RGP were identified. These lines were considered as potential donors for pyramiding superior CG haplotypes for improving PUE in rice.

## 3 Results

### 3.1 Features of the QTL studies used in MQTL analysis

**Figure 1:**
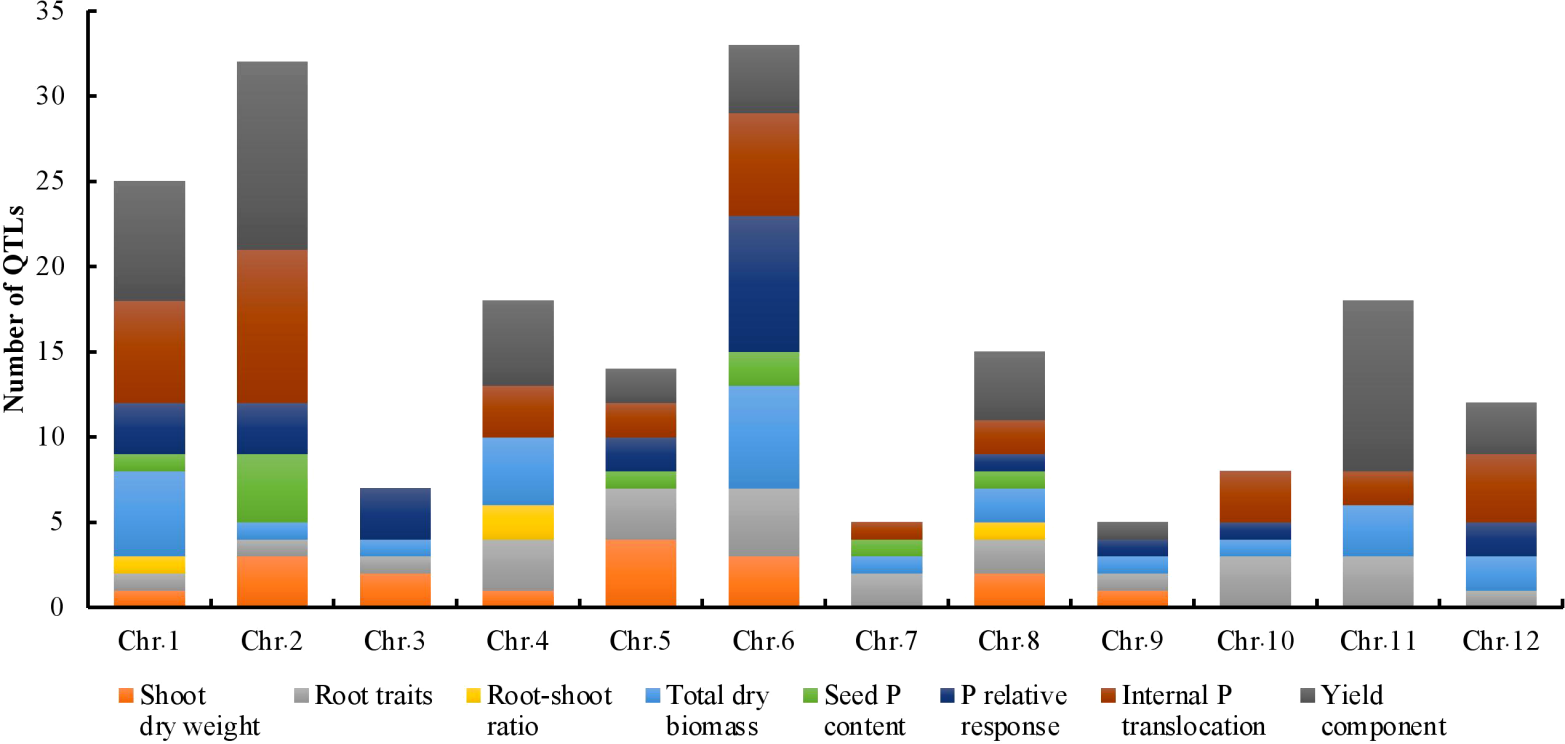
Phenotypic trait classes and chromosome-wise distribution of QTLs utilized in the MQTL analysis for PUE in rice.

**Figure 2:**
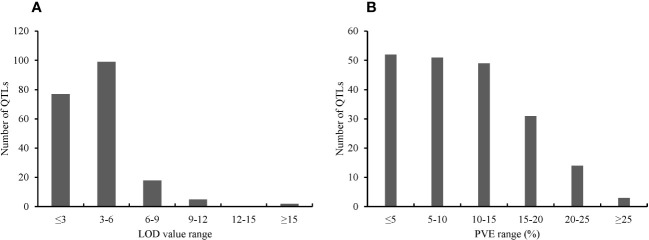
Features of the PUE QTLs used in the MQTL analysis. **(A)** Distribution of LOD scores **(B)** Distribution of PVE values of the initial PUE.

The key features of the PUE QTL studies used in the PUE MQTL analysis are presented in (**Table 1**). A total of 192 QTLs from 16 independent studies published between 1998 and 2022 were used to identify PUE MQTLs. These QTLs were mapped from 15 non-overlapping bi-parental populations, including CSSLs (chromosome segment substitution lines) (3), DH (doubled haploids) (2), F_2_ (2), RILs (recombinant inbred lines) (5), F_2:3_ (F_2_-derived F_3_) (2), and BILs (backcross inbred lines) (3). The markers employed in mapping PUE QTLs included SNP (single nucleotide polymorphism) (6); AFLP (amplified fragment length polymorphism) (2); SSR (simple sequence repeat) (6); RFLP (restriction fragment length polymorphism) (4); and InDel (insertion/deletion polymorphism) (1). The numbers of the initial QTLs per trait group were as follows: SDW (n=18); RT (n=25); RSR (n=4); BM (n=31); SPC (n=10); RRP (n=24); PUE (n=36); YLD (n=44). The distribution of the initial QTLs varied widely across chromosomes (**Figure 1**). There were largest numbers of QTLs located on chromosomes 2 (n=32) and 6 (n=33). On the other hand, only seven and five QTLs were located on chromosomes 3 and 9, respectively. The PVE values and LOD scores had ranges of 1.25%~27.9% and 2.0~16.98, respectively. More than half (51%) of the PVE values were between 3 and 6% (**Figure 2A**), whereas most of the LOD scores were less than five (**Figure 2B**).

### 3.2 The rice consensus map and QTL projection

**Figure 3:**
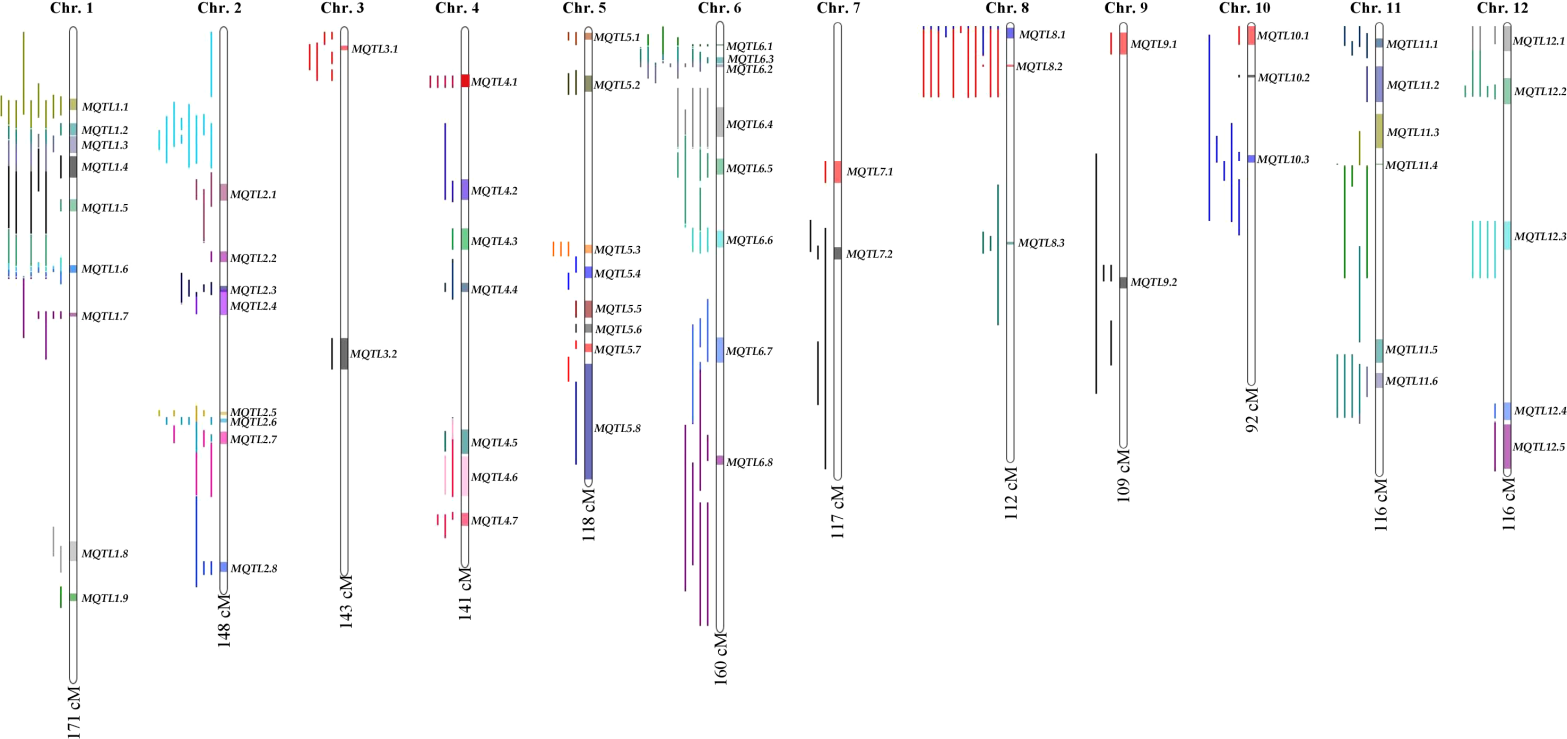
PUE MQTLs identified across 12 rice chromosomes.

The consensus map included a total of 5,694 markers, comprising both SNPs from the 6K Infinium SNP array and the flanking markers of the QTLs used in the MQTL analysis. They were well-distributed across the twelve chromosomes. The SNP density per 500 kb window is presented in (**Figure S1**). In addition, the positions of known PUE genes, such as *OsPSTOL1*, *OsPTF1*, *OsERF3*, and *OsTHS1*, were incorporated onto the consensus map. The cumulative length of the consensus map was 1,637 cM. The average distance between the adjacent markers was 0.29 cM. Chromosomes 1 (171.9 cM) and 6 (160 cM) were the longest, while chromosomes 9 (108.5 cM) and 10 (92.2 cM) were the shortest. The number of markers varied from 332 to 653 per chromosome. Chromosomes 1 and 2 had the highest counts of 653 and 574 markers, respectively, while chromosomes 9 (n=354) and 10 (n=332) were the least saturated. We projected the PUE QTLs onto the consensus map using both the physical position of the QTLs and the SNP markers (**Figure 3**). The number of QTLs ranged from 5 to 33. Chromosomes 6 (n=33), 2 (n=32), and 1 (n=25) were the most saturated regions, while chromosomes 9 (n=5), 7 (n=5), and 3 (n=7) had the fewest.

### 3.3 MQTL analysis

**Figure 4:**
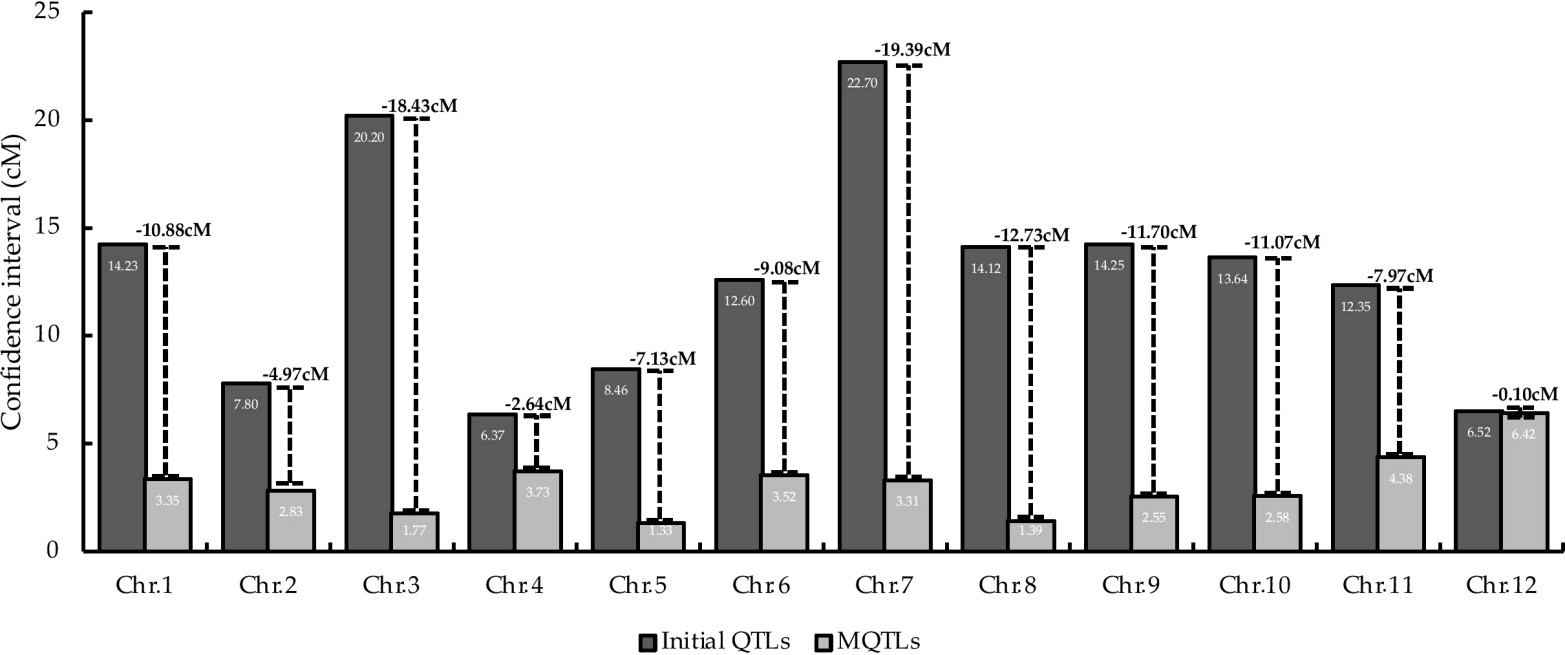
Comparison of average CI between initial the QTLs and MQTLs on the 12 rice chromosomes. Values on top of the lines represent the reduction in the average CI.

MQTL analysis of the 192 initial PUE QTLs resulted in the identification of 60 MQTLs (**Table 2**; **Figure 3**). The distribution of MQTLs across chromosomes was uneven. The highest number of MQTLs were detected on chromosomes 1 (n=9) and 5 (n=8), while chromosomes 3 (n=2), 7 (n=2), and 9 (n=2) had the least. Reductions in MQTL CI were observed, with fold reductions ranging from 2.8 to 11.4 with an overall average reduction of 9.7 cM (**Figure 4**). The CI for MQTLs located on chromosomes 3, 5, 7, 9, and 10 were at least five times smaller than their corresponding initial QTLs, while those on chromosomes 1, 2, 6, and 11 were at least twice as small. Chromosomes 4 and 12 exhibited the least reduction in CI, with fold change values of 1.7 and 1.02, respectively. The MQTLs had PVE values from 2.3 to 20.3.

**Table 2:** Number of initial QTLs and MQTLs identified on rice chromosomes.

Chromosome	No. of the Initial QTLs^a^	No. of MQTLs
1	25	9
2	32	8
3	7	2
4	18	7
5	14	8
6	33	5
7	5	2
8	15	3
9	5	2
10	8	3
11	18	6
12	12	5
**Total**	**192**	**60**

^**a**^ **Initial QTLs:** QTLs used for MQTL analysis.

Subsequently, we selected MQTLs that had an average PVE value of at least five and were supported by QTLs from at least two independent studies (**Table 3**). This resulted in 38 MQTLs, with the number of QTL members ranging from 2 to 11. MQTL1.1, MQTL8.1, and MQTL8.2 had the most QTLs (n=11), while MQTL3.1, MQTL4.1, and MQTL10.1 had only two initial QTLs. The MQTLs had CI ranging from 0.2 to 9 cM, with an average of 3.5 cM per MQTL. The smallest CI (0.2~2 cM) were observed on MQTL1.7, MQTL2.1, MQTL3.1, MQTL4.5, MQTL5.1, MQTL6.1, MQTL6.2, MQTL6.3, MQTL8.2, MQTL8.3, MQTL10.2, MQTL10.3, and MQTL11.4, while the largest CI (7~9 cM) were observed on MQTL6.7, MQTL6.4, MQTL11.3, MQTL12.1, and MQTL12.2. The initial QTL members per MQTL varied from two to six per MQTL. MQTL1.5, MQTL1.6, MQTL1.7, and MQTL6.4 had the greatest number of initial QTL at n=6. Interestingly, MQTL2.1 had only one initial QTL (RRP). MQTL1.5, MQTL1.6, and MQTL1.7 had the colocalizing QTLs underlying BM, SCP, RSR, PUE, YLD, and RRP. Initial QTLs modulating BM, RRP, PUE, SPC, and YLD were all collocated on MQTL6.1, MQTL6.2, and MQTL6.3. YLD and BM were the most abundant traits in the MQTLs, present in 66% and 61% of the total MQTLs, respectively. In contrast, RSR was the least abundant trait, present in only 23% of the MQTLs.

**Table 3:** Summary of rice PUE MQTLs supported by multiple QTLs from at least two independent studies and with at least 5% average PVE values.

MQTL	Chromosome	AIC value (Model) ^a^	No. of QTLs involved	Initial QTL average PVE (%)	Traits Involved ^b^	Position (cM)	95% CI^c^. (cM)	Start (cM)	Stop (cM)	Left Marker	Right Marker	Physical Position (Mb)	Number of annotated genes underlying MQTL
*MQTL1.1*	1	230.18 (10)	11	12.3	YLD, BM, RRP, SPC, RSR, BM	19.51	3.10	17.96	21.06	SNP144359	SNP163177	4.49 – 5.17	85
*MQTL1.2*	1		7	13.0	BM, YLD, SPC, RSR	26.14	3.39	24.45	27.84	SNP193627	id1005232	6.13 – 6.9	104
*MQTL1.3*	1		7	12.5	PUP, YLD, BM, SPC, RSR	30.25	4.55	27.98	32.53	SNP222467	SNP245712	7.12 – 7.92	127
*MQTL1.4*	1		7	12.3	YLD, BM, SPC, RSR, IPT, BM	36.07	5.79	33.18	38.97	SNP255699	SNP298163	8.30 – 9.88	217
*MQTL1.5*	1		6	8.9	YLD, BM, SPC, RSR, IPT, RRP	46.33	3.29	44.69	47.98	SNP337593	SNP368391	11.16 – 11.94	82
*MQTL1.6*	1		7	8.3	BM, SPC, RSR, IPT, YLD, RRP	63.29	2.12	62.23	64.35	SNP513565	SNP528131	15.57 – 16.03	32
*MQTL1.7*	1		5	9.3	BM, SPC, RSR, IPT, YLD, RRP	75.62	1.20	75.02	76.22	SNP634609	SNP642207	18.89 – 19.09	22
*MQTL2.1*	2	278.41 (3)	3	9.5	RRP	44.31	1.09	43.77	44.86	id2005182	SNP1717430	11.00 – 11.23	24
*MQTL2.7*	2		7	11.7	SPC, BM, IPT, RT	105.31	4.57	103.03	107.60	id2011296	SNP2252638	25.77 – 26.88	159
*MQTL3.1*	3	35.98 (4)	2	9.3	BM, RT	0.88	1.77	0.02	1.79	SNP2491701	SNP2492869	0.40 – 0.47	8
*MQTL4.1*	4	124.61 (4)	2	9.1	RT, BM	31.98	6.60	28.68	35.28	SNP3933751	SNP3993698	7.17 – 8.84	118
*MQTL4.3*	4		3	8.5	RT, YLD	73.19	2.47	71.96	74.43	id4009413	id4010200	28.56 – 30.27	272
*MQTL4.4*	4		3	10.0	SDW, YLD	99.15	5.63	96.34	101.97	SNP4511040	SNP4543652	24.10 – 25.44	246
*MQTL4.5*	4		3	8.0	BM, IPT, YLD	127.50	0.20	127.40	127.60	SNP4715080	id4010985	31.78 – 32.04	46
*MQTL5.1*	5	144.34 (8)	3	7.0	SPC, RT	1.33	1.92	0.37	2.29	id5000043	SNP4821710	0.10 – 0.63	96
*MQTL6.1*	6	305.93 (5)	7	10.7	BM, RRP, IPT, SPC, YLD	5.63	0.50	5.38	5.88	id6000911	SNP5865517	1.38 – 1.42	9
*MQTL6.2*	6		9	6.5	BM, RRP, IPT, SPC, YLD	9.62	1.71	8.77	10.48	SNP5883472	SNP5895767	2.13 – 2.61	108
*MQTL6.3*	6		8	8.9	BM, RRP, IPT, SPC, YLD	10.98	0.82	10.57	11.39	SNP5895767	SNP5903052	2.61 – 2.87	47
*MQTL6.4*	6		6	20.4	SDW, RRP, IPT, SPC, YLD, RT	26.18	8.12	22.12	30.24	SNP5980679	SNP6051078	5.71 – 7.58	278
*MQTL6.5*	6		5	13.0	RT, YLD, BM	37.87	4.42	35.66	40.08	id6005608	SNP6147112	8.73 – 10.2	138
*MQTL6.6*	6		5	13.5	BM, RT	57.20	4.68	54.86	59.54	SNP6294233	SNP6351040	13.73 – 14.94	62
*MQTL6.7*	6		5	13.3	RRP, BM	86.61	6.91	83.16	90.07	SNP6613416	SNP6686237	20.83 – 22.52	179
*MQTL6.8*	6		7	14.2	RRP, BM, RT	116.04	2.61	114.74	117.35	SNP6889734	SNP6906770	28.69 – 29.33	95
*MQTL7.2*	7	34.17 (3)	3	9.3	PUP, SPC	58.54	3.31	56.89	60.20	SNP7468473	id7002392	14.11 – 15.01	57
*MQTL8.1*	8	95.18 (3)	11	8.5	YLD, SPC, BM, RSR, RT	1.87	2.80	0.47	3.27	id8001299	SNP8007342	0.12 – 0.82	126
*MQTL8.2*	8		11	15.1	YLD, SPC, BM, RSR, RT	10.43	0.68	10.09	10.77	id8002025	wd8001250	2.52 – 2.69	7
*MQTL8.3*	8		4	9.0	IPT, RRP	56.46	0.70	56.11	56.81	SNP8545780	SNP8550504	14.05 – 14.18	10
*MQTL9.2*	9	37.81 (4)	4	13.0	YLD, RRP	54.08	2.55	52.81	55.36	SNP9600918	SNP9623212	13.15 – 13.79	53
*MQTL10.1*	10	48.96 (4)	2	9.4	IPT, RT	2.45	4.90	0.00	4.90	SNP9898598	SNP9941068	0.15 – 1.01	70
*MQTL10.2*	10		3	11.5	IPT, RRP	13.07	0.78	12.68	13.46	id10000881	SNP10063204	3.02 – 3.4	21
*MQTL10.3*	10		7	9.2	RRP, IPT, SDW, RT	34.54	2.05	33.51	35.56	SNP10309364	id10002487	8.40 – 8.83	19
*MQTL11.1*	11	148.94 (6)	5	6.3	RT, YLD, IPT	4.52	2.48	3.28	5.76	SNP10840785	SNP10859595	0.81 – 1.42	94
*MQTL11.3*	11		4	14.0	IPT, RT, YLD	27.36	9.00	22.86	31.86	SNP10987441	SNP11075456	5.70 – 7.86	239
*MQTL11.4*	11		6	14.0	YLD, RT, RRP	36.00	0.33	35.84	36.17	id11005058	id11007108	15.45 – 19.35	355
*MQTL11.5*	11		4	9.8	YLD, IPT, BM, RT	84.47	6.17	81.39	87.56	SNP11608239	SNP11688144	17.69 – 19.18	202
*MQTL11.6*	11		6	14.4	YLD, IPT, BM, RT	92.03	3.90	90.08	93.98	SNP11730518	SNP11760343	21.49 – 21.84	77
*MQTL12.1*	12	95.86 (5)	4	6.0	YLD, RRP	2.33	8.45	0.00	6.56	SNP12006654	SNP12045914	0.12 – 1.64	303
*MQTL12.2*	12		6	11.0	BM, YLD, RRP	16.99	6.90	13.54	20.44	SNP12088643	id12002348	3.14 – 5.13	250

^**a**^ For the model number in the parentheses of AIC value, see the materials and methods.

^**b**^ **SDW**, shoot dry weight; **RT**, absolute value of root traits; **RSR,** root-shoot ratio; **BM**, total dry biomass; **SPC**, seed P content; **RRP**, relative response to P; **IPT**, internal P translocation; **YLD**, yield component.

^**c**^ **CI,** Confidence interval.

### 3.4 Identification of CGs underlying PUE in rice and functional analysis

The MQTLs harbored a total of 4,370 non-redundant genes. The number of genes per MQTL varied widely, from seven to 355, with MQTL11.4 (n=355) and MQTL12.1 (n=303) having the highest number of genes, while MQTL6.1 and MQTL8.2 having the fewest genes involved (seven and nine genes, respectively) (**Table 3**). After filtering for PUE-related terms, we identified 273 CGs (**Table S1**), of which 238 had root expression data available in the RiceXPro database (**Table S2**). Further analysis of CGs revealed that 209 genes had consistent direction of regulation under P non-supplied condition (184 upregulated, 25 downregulated) across the two time-points (6h and 24h) post-treatment (**Table S3**). The number of CGs had a strong positive correlation (*R^2^ = *64%) with CI (**Figure S3**), with exceptions on some MQTLs. In some MQTLs with small CI values, high number of PUE CGs were investigated, say, MQTL2.7 (CI=4.57 cM, n CGs=24), MQTL4.3 (CI=2.7 cM, n CGs=31), MQTL11.4 (CI=0.33 cM, n CGs=16). In contrast, MQTL4.1 (CI=6.6 cM, n CGs=4), MQTL10.1 (CI=4.9 cM, n CGs=1), MQTL11.3 (CI=9 cM, n CGs=7) had a relatively larger CI but fewer CGs.

**Figure 5:**
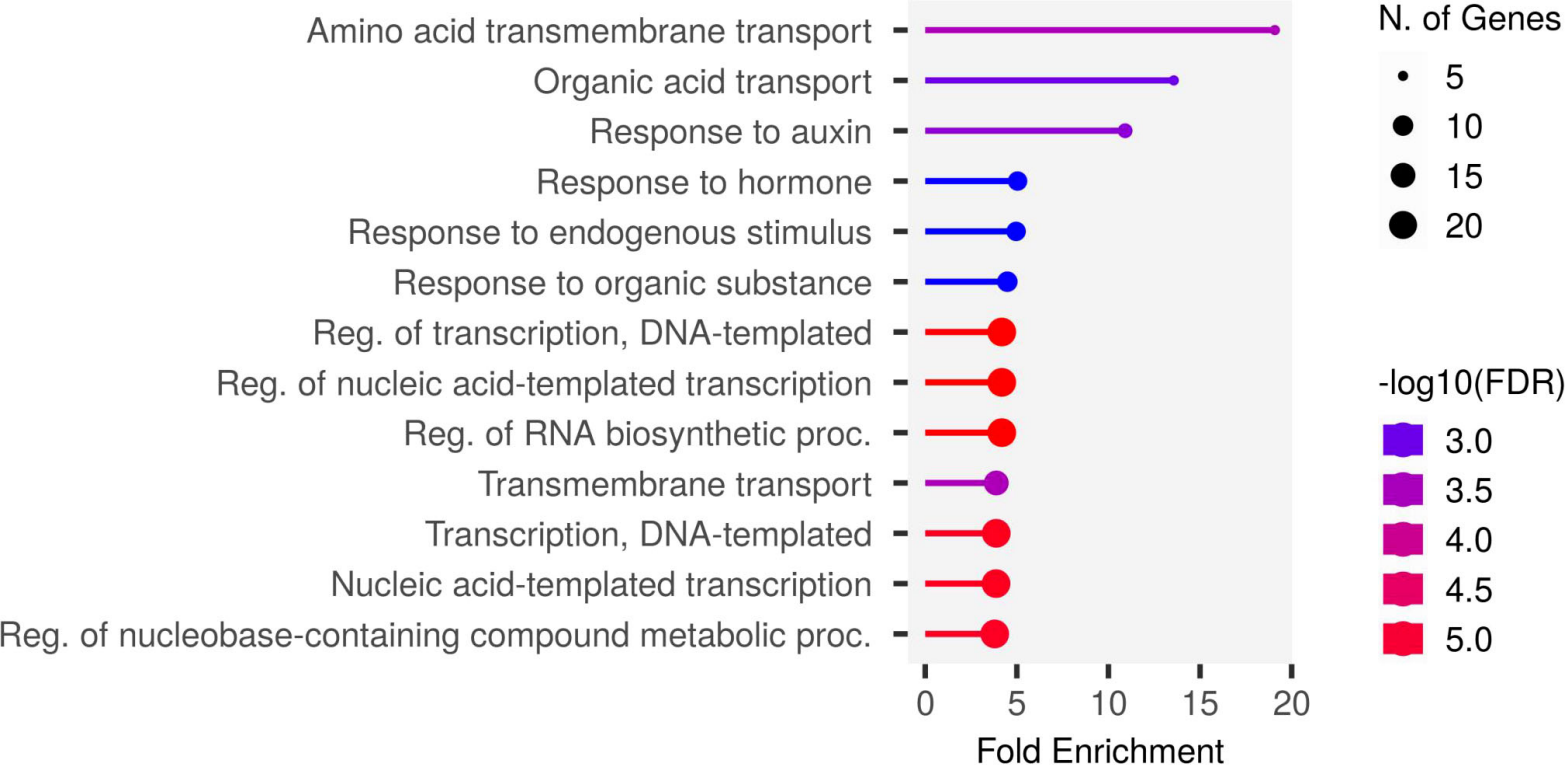
The most enriched biological pathways in the PUE CGs.

**Figure 6:**
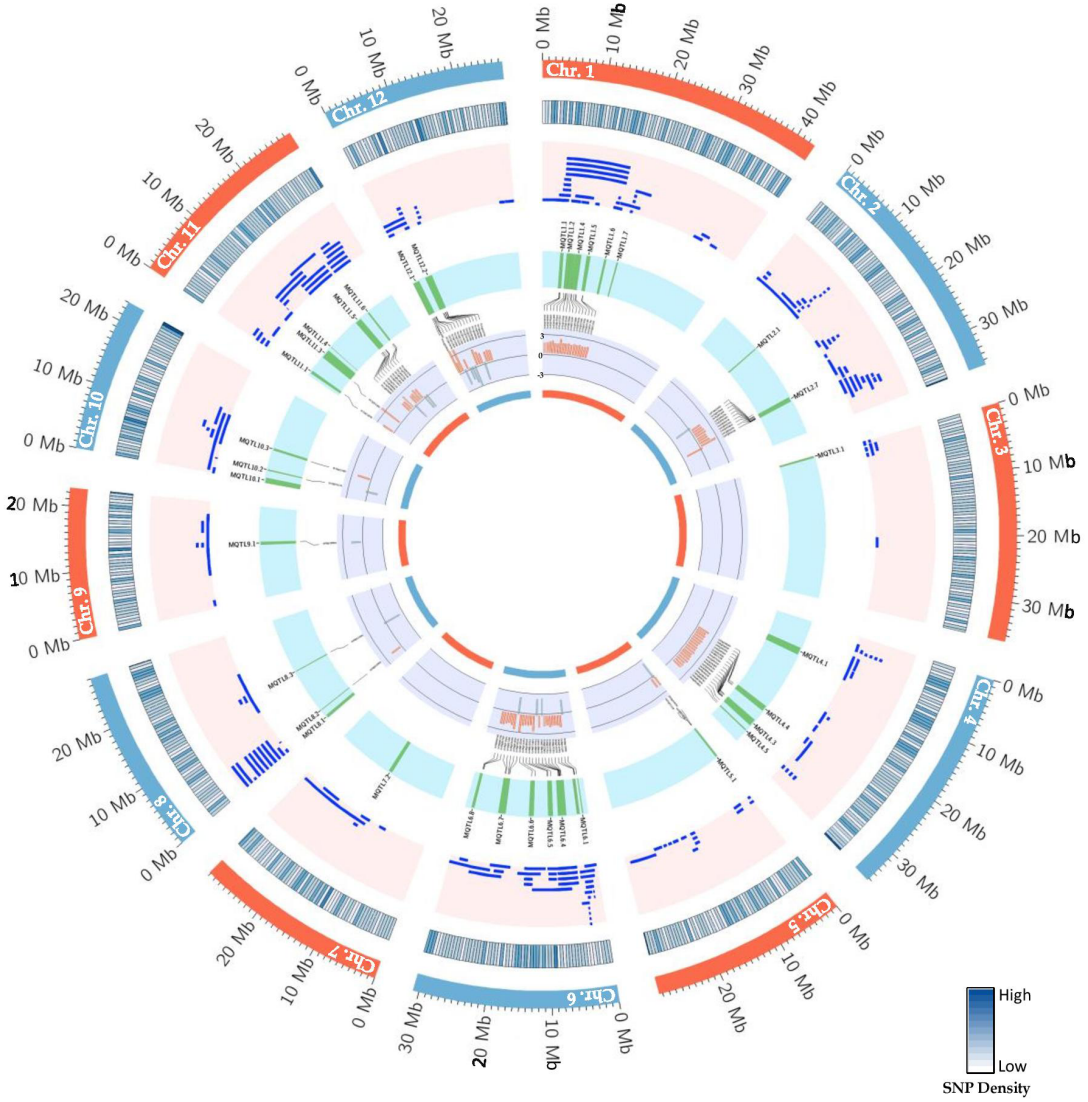
Schematic representation of the distribution patterns of PUE QTLs, MQTLs, and PUE CGs on rice chromosomes. The outermost circle represents the rice karyotype. The second circle outlines the consensus map SNP density (500kb window). The third circle display the initial PUE QTLs used in the MQTL analysis. The fourth inner circle represents the MQTLs supported by QTLs from at least 2 independent studies and with a PVE value of ≥ 5%. The fifth and sixth inner circle represent the P-responsive CGs and the fold change in gene expression (P non-supplied vs. control treatment), respectively.

To understand the biological pathways involved, we conducted gene ontology (GO) enrichment analysis using the 103 PUE CGs. Amino acid transmembrane transport, organic transport, and response to auxin were the most enriched biological pathways with five, five, and six gene members, respectively. Transcription, DNA templated, nucleic acid-templated transcription, and regulation of nucleobase-containing compound metabolic process had the most gene members (20, 21, and 21, respectively) (**Figure 5**). To identify genes that are responsive to P treatment, we set a threshold of a 1.5-fold change in expression (P non-supplied condition vs. control), which narrowed down the list of CGs to 103 (**Table 4**), of which 86 were upregulated and 17 were downregulated (**Figure 6**).

**Table 4:** P-responsive candidate genes underlying PUE MQTLs supported by multiple QTLs from at least two independent studies and with at least 5% average PVE values.

MQTL	MSU ID	RAP ID	Symbol	Description
*MQTL1.1*	*LOC_Os01g09550*	*Os01g0191300*	*ONAC003*	Similar to NAC-type transcription factor;NAC transcription factor, Drought resistance.
	*LOC_Os01g09700*	*Os01g0192900*	*OsACS5*	1-aminocyclopropane-1-carboxylic acid synthase, Submergence response.
	*LOC_Os01g09850*	*Os01g0195000*	*OsIDD2*	Zinc finger and indeterminate domain (IDD) family transcription factor, Regulation of secondary cell wall formation.
*MQTL1.2*	*LOC_Os01g11520*	*Os01g0213400*	*OsRFPH2-8*	Zinc finger, RING/FYVE/PHD-type domain containing protein.
	*LOC_Os01g12260*	*Os01g0222500*	*OsVHA-E3*	Similar to Vacuolar ATP synthase subunit E (EC 3.6.3.14) (V-ATPase E subunit) (Vacuolar proton pump E subunit).
	*LOC_Os01g12400*	*Os01g0223700*	*SDRLK-68*	S-Domain receptor like kinase-68, Partial S-domain containing protein, Response to drought in tolerant rice genotypes
	*LOC_Os01g12440*	*Os01g0224100*	*OsERF#053*	Similar to DNA binding protein-like protein; Similar to DNA binding protein-like protein.
*MQTL1.3*	*LOC_Os01g13030*	*Os01g0231000*	*OsIAA3*	Similar to Auxin-responsive protein (Aux/IAA) (Fragment). Similar to Auxin-responsive protein (Aux/IAA) (Fragment).
	*LOC_Os01g13770*	*Os01g0239200*	*OsTPT1*	Triose phosphate/phosphate translocator
*MQTL1.4*	*LOC_Os01g14870*	*Os01g0252200*	*OsC3H3*	Zinc finger, CCCH-type domain containing protein.
	*LOC_Os01g15300*	*Os01g0256800*	*OsC3H4*	Similar to zinc finger helicase family protein. Hypothetical conserved gene.
	*LOC_Os01g16260*	*Os01g0268100*	*OsZIFL1*	Similar to Major facilitator superfamily antiporter.
	*LOC_Os01g16940*	*Os01g0276500*	–	Similar to Histidine biosynthesis bifunctional protein hisIE, chloroplast precursor; Phosphoribosyl-ATP pyrophosphatase (EC 3.6.1.31) (PRA-PH)].
	*LOC_Os01g17240*	*Os01g0279700*	*OsPT21*	Phosphate transporter 4;1;Major facilitator superfamily protein.
*MQTL1.5*	*LOC_Os01g20930*	*Os01g0311400*	*OsRING108*	Zinc finger, *RING/FYVE/PHD*-type domain containing protein.
	*LOC_Os01g20940*	*Os01g0311500*	*OsPHS1a*	Similar to *PHS1* (PROPYZAMIDE-HYPERSENSITIVE 1); phosphoprotein phosphatase/protein tyrosine/serine/threonine phosphatase.
	*LOC_Os01g21120*	*Os01g0313300*	*OsERF#068*	Similar to *EREBP-3* protein (Fragment).
*MQTL2.7*	*LOC_Os02g41800*	*Os02g0628600*	*OsARF8*	Similar to Auxin response factor 8.
	*LOC_Os02g42690*	*Os02g0639800*	*OsRDCP2*	Hypothetical gene. Zinc finger, *RING/FYVE/PHD*-type domain containing protein.
	*LOC_Os02g42990*	*Os02g0643800*	*OsSAUR11*	Auxin-responsive protein, Negative regulation of deep sowing tolerance, Mesocotyl elongation.
	*LOC_Os02g43170*	*Os02g0646200*	*OsBBX6*	Zinc finger, B-box domain containing protein.
	*LOC_Os02g43620*	*Os02g0652800*	*OsGlpT1*	Major facilitator superfamily *MFS*_1 protein.
	*LOC_Os02g43790*	*Os02g0654700*	*OsERF#091*	*AP2/ERF* family protein, Abiotic stress response.
	*LOC_Os02g43820*	*Os02g0655200*	*OsERF#095*	Similar to Ap25. ERF family protein, Transcriptional regulator, Salt stress tolerance.
	*LOC_Os02g43940*	*Os02g0656600*	*OsERF#032*	Similar to Dehydration responsive element binding protein 2B (DREB2B protein).
	*LOC_Os02g44090*	*Os02g0658200*	–	Zinc finger, PHD-type domain containing protein.
	*LOC_Os02g44120*	*Os02g0659100*	*OsDLN62*	Zinc finger, C2H2-type domain containing protein.
*MQTL4.4*	*LOC_Os04g41350*	*Os04g0490900*	*OsAAP11G*	Similar to *OSIGBa0130B08.4* protein.
	*LOC_Os04g42090*	*Os04g0498600*	*SamDC*	S-adenosylmethionine decarboxylase, Polyamine biosynthesis, Salt and drought stresses, Abiotic stress.
	*LOC_Os04g42570*	*Os04g0504500*	*OsPLT4*	Similar to protein *BABY BOOM 1.*
*MQTL4.3*	*LOC_Os04g48050*	*Os04g0568900*	*OsRINGzf1*	RING zinc finger protein, E3 ubiquitin ligase, Regulation of drought resistance.
	*LOC_Os04g48170*	*Os04g0570000*	*OsCYP87A3*	Cytochrome P450 87A3, Auxin signaling in the regulation of coleoptile growth.
	*LOC_Os04g48410*	*Os04g0573200*	*SOD*	Copper chaperone for superoxide dismutase, Target of miR398b, Resistance to rice blast disease;Similar to OSIGBa0147H17.7 protein.
	*LOC_Os04g49000*	*Os04g0579200*	*OsRING328*	Zinc finger, *RING/FYVE/PHD*-type domain containing protein.
	*LOC_Os04g49410*	*Os04g0583500*	*OsEXPA10*	Expansin, Al-inducible expansin, Root cell elongation;Similar to Expansin-A10.
	*LOC_Os04g49510*	*Os04g0584600*	*OsCDPK13*	Similar to Calcium dependent protein kinase. Group I calcium-dependent protein kinase, Cold and salt/drought tolerance.
	*LOC_Os04g49620*	*Os04g0585700*	*OsFLZ12*	Protein of unknown function DUF581 family protein.
	*LOC_Os04g49650*	*Os04g0585900*	*OsFLZ13*	Protein of unknown function DUF581 family protein. Protein of unknown function DUF581 family protein.
	*LOC_Os04g49660*	*Os04g0586000*	*OsFLZ14*	Protein of unknown function DUF581 family protein.
	*LOC_Os04g49670*	*Os04g0586100*	*OsFLZ15*	Protein of unknown function DUF581 family protein.
	*LOC_Os04g49680*	*Os04g0586200*	*OsFLZ16*	Similar to H0307D04.13 protein.
	*LOC_Os04g51890*	*Os04g0608300*	*OsSAUR20*	Auxin responsive SAUR protein domain containing protein.
	*LOC_Os04g52900*	*Os04g0620000*	*OsABCC1*	C-type ATP-binding cassette (ABC) transporter, Arsenic (As) detoxification, Reduction of As in grains.
*MQTL4.5*	*LOC_Os04g53612*	*Os04g0628000*	*OsISC40*	Protein of unknown function DUF794, plant family protein.
*MQTL5.1*	*LOC_Os05g01610*	*Os05g0106700*	*OsPRAF2*	Similar to PRAF1; Ran GTPase binding/chromatin binding/zinc ion binding.
	*LOC_Os05g01990*	*Os05g0110500*	*OsRH17*	Similar to DEAD-box ATP-dependent RNA helicase 17. DEAD-box RNA helicase protein, Stress responses.
	*LOC_Os05g02050*	*Os05g0111100*	–	Zinc finger, Tim10/DDP-type family protein.
*MQTL6.1*	*LOC_Os06g03860*	*Os06g0129400*	*OsSPX-MFS3*	Splicing variant of *SPX-MFS* protein 3. Vacuolar phosphate efflux transporter, Pi homeostasis.
*MQTL6.2*	*LOC_Os06g04920*	*Os06g0141200*	*OsZFP1*	Putative zinc finger protein, Negative regulation of salt stress response.
	*LOC_Os06g05110*	*Os06g0143000*	*SodB*	Iron-superoxide dismutase;Splicing variant of the iron-superoxide dismutase.
	*LOC_Os06g05160*	*Os06g0143700*	*OsSultr3;4*	*SULTR*-like phosphorus distribution transporter, Control of the allocation of phosphorus to the grain.
*MQTL6.4*	*LOC_Os06g11450*	*Os06g0218300*	*OsRING342*	Zinc finger, *RING*-type domain containing protein.
	*LOC_Os06g11860*	*Os06g0222400*	*OsERF#120*	Similar to *DRE*-binding protein 2.
	*LOC_Os06g11980*	*Os06g0223700*	*OsFLZ20*	*FCS*-like zinc finger (FLZ) protein 20, Submergence response.
	*LOC_Os06g12160*	*Os06g0225900*	–	Similar to ATP binding/ATPase/nucleoside-triphosphatase/nucleotide binding.
	*LOC_Os06g12370*	*Os06g0229000*	*OsFtsH6*	Similar to FtsH protease (VAR2) (Zinc dependent protease).
	*LOC_Os06g12610*	*Os06g0232300*	*PIN1C*	PIN protein, Auxin efflux carrier, Auxin transport and signaling, “Root, shoot and inflorescence development”.
	*LOC_Os06g12640*	*Os06g0232700*	–	Similar to SWIM zinc finger family protein.
*MQTL6.5*	*LOC_Os06g16060*	*Os06g0271600*	*OsRING141*	Zinc finger, *RING/FYVE/PHD*-type domain containing protein.
	*LOC_Os06g17280*	*Os06g0283200*	*OsRFP*	Zinc finger, *RING/FYVE/PHD*-type domain containing protein.
	*LOC_Os06g17410*	*Os06g0284500*	*OsDof20*	Zinc finger, Dof-type family protein.
*MQTL6.6*	*LOC_Os06g23530*	*Os06g0343100*	–	Similar to ATP-dependent helicase DHX8 (RNA helicase HRH1) (DEAH-box protein 8). Similar to predicted protein.
	*LOC_Os06g24850*	*Os06g0355300*	*OsIAA22*	Similar to Auxin-responsive protein IAA22.
*MQTL6.7*	*LOC_Os06g35960*	*Os06g0553100*	*HSfC2b*	Similar to Heat stress transcription factor C-2b.
	*LOC_Os06g36210*	*Os06g0556200*	*OsAAP12B*	Similar to amino acid permease 1.
	*LOC_Os06g36560*	*Os06g0561000*	*OsMIOX*	Myo-inositol oxygenase, Drought stress tolerance.
	*LOC_Os06g37450*	*Os06g0571800*	*OsGATA16*	Similar to GATA transcription factor 20. Similar to GATA transcription factor 3 (AtGATA-3).
	*LOC_Os06g37750*	*Os06g0575400*	*SDRLK-5*	S-Domain receptor like kinase-5, Response to drought in a tolerant genotype, Response to submergence.
*MQTL6.8*	*LOC_Os06g47290*	*Os06g0687400*	–	Similar to auxin-independent growth promoter-like protein. (Os06t0687400-01);Similar to auxin-independent growth promoter-like protein.
	*LOC_Os06g47590*	*Os06g0691100*	*OsERF#121*	Pathogenesis-related transcriptional factor and ERF domain containing protein.
	*LOC_Os06g47840*	*Os06g0693500*	–	Zinc finger, C2H2-like domain containing protein.
*MQTL8.2*	*LOC_Os08g05030*	*Os08g0145600*	–	Similar to cDNA clone:J023091L02, full insert sequence.
	*LOC_Os08g23410*	*Os08g0323400*	*OsISC42*	Similar to Rubredoxin (Rd).
*MQTL9.1*	*LOC_Os09g23140*	*Os09g0394600*	–	Endonuclease/exonuclease/phosphatase domain containing protein.
*MQTL10.2*	*LOC_Os10g06030*	*Os10g0151100*	*OsWAK103*	Similar to Protein kinase domain containing protein, expressed.
*MQTL10.3*	*LOC_Os10g16974*	*Os10g0317900*	*OsCYP75B4*	Chrysoeriol 5’-Hydroxylase, Flavonoid B-ring hydroxylase, Tricin biosynthesis; Similar to Flavonoid 3-monooxygenase.
*MQTL11.1*	*LOC_Os11g03420*	*Os11g0128300*	*OsMIF1*	Mini zinc finger protein, A member of the *ZF-HD* (zinc finger-homeodomain) family, Negative regulation of deep sowing tolerance, Mesocotyl elongation.
*MQTL11.3*	*LOC_Os11g10590*	*Os11g0211800*	*OsDT11*	Cysteine-rich peptide, Short-chain peptide, ABA-dependent drought tolerance.
*MQTL11.4*	*LOC_Os11g29870*	*Os11g0490900*	*OsWRKY72*	*WRKY* transcription factor 72, ABA response with respect to germination and abiotic stresses, ABA signaling and auxin transport
	*LOC_Os11g30484*	*Os11g0498400*	–	Zinc finger, C2H2-like domain containing protein.
	*LOC_Os11g30560*	*Os11g0499600*	*drp7*	Hydroxysteroid dehydrogenase, Cuticle formation, Lipid homeostasis, Submergence tolerance.
	*LOC_Os11g31340*	*Os11g0512100*	*ONAC127*	NAC (NAM, ATAF1/2, CUC2) transcription factor, Heat stress response, Regulation of grain filling.
	*LOC_Os11g32100*	*Os11g0523700*	*OsbHLH002*	bHLH transcription factor, Positive regulation of chilling tolerance, Control of stomatal initiation, Regulation of mature stoma differentiation.
	*LOC_Os11g32110*	*Os11g0523800*	*OsARF1*	Similar to Isoform 3 of Auxin response factor 23. Auxin response factor 1.
	*LOC_Os11g32290*	*Os11g0525900*	–	Zinc finger, GRF-type domain containing protein.
*MQTL11.6*	*LOC_Os11g36480*	*Os11g0573200*	–	Similar to Zinc knuckle family protein, expressed.
	*LOC_Os11g36960*	*Os11g0578100*	*OsDjC76*	Heat shock protein DnaJ, N-terminal domain containing protein.
	*LOC_Os11g37000*	*Os11g0578500*	*OsDjC77*	Heat shock protein DnaJ family protein.
*MQTL12.1*	*LOC_Os12g01530*	*Os12g0106000*	*OsFER2*	Ferritin, Iron storage protein, Iron homeostasis.
	*LOC_Os12g02100*	*Os12g0112300*	–	ADP/ATP carrier protein domain containing protein.
	*LOC_Os12g02450*	*Os12g0116700*	*OsWRKY64*	*WRKY* transcription factor 64, Response to the rice pathogens, Regulation of root elongation under iron excess, Iron stress tolerance.
	*LOC_Os12g02980*	*Os12g0123500*	–	Similar to Apyrase precursor (EC 3.6.1.5) (ATP-diphosphatase) (Adenosine diphosphatase) (ADPase) (ATP-diphosphohydrolase).
	*LOC_Os12g03670*	*Os12g0130500*	*SDRLP-6*	S-Domain receptor like protein-6, Response to submergence.
	*LOC_Os12g03830*	*Os12g0132500*	*OsZIFL9*	Similar to Major facilitator superfamily antiporter.
	*LOC_Os12g03950*	*Os12g0133300*	*OsZIFL13*	Similar to Carbohydrate transporter/sugar porter/transporter.
*MQTL12.2*	*LOC_Os12g07700*	*Os12g0176200*	*OsISC14*	Similar to Nitrogen fixation like protein.
	*LOC_Os12g08070*	*Os12g0181300*	–	Similar to TRAF-type zinc finger family protein.
	*LOC_Os12g08090*	*Os12g0181500*	*OsAAP11A*	Amino acid permease, Transport of amino acids.
	*LOC_Os12g08130*	*Os12g0181600*	*OsAAP11B*	Amino acid transporter, transmembrane domain containing protein.
	*LOC_Os12g08780*	*Os12g0189500*	*OsYUCCA11*	Flavin-containing monooxygenase, Auxin biosynthesis, Endosperm development, Regulation of grain filling.
	*LOC_Os12g08820*	*Os12g0190100*	–	Similar to Auxin-independent growth promoter-like protein.
	*LOC_Os12g09300*	*Os12g0194900*	*OsAAP10B*	Amino acid permease, A member of the amino acid transporter (AAT) family, Regulation of tillering and grain yield, Regulation of neutral amino acid transport
	*LOC_Os12g09590*	*Os12g0197700*	–	Region of unknown function, putative Zinc finger, XS and XH domain containing protein.

### 3.5 Haplotype analysis and identification of potential donors

**Figure 7:**
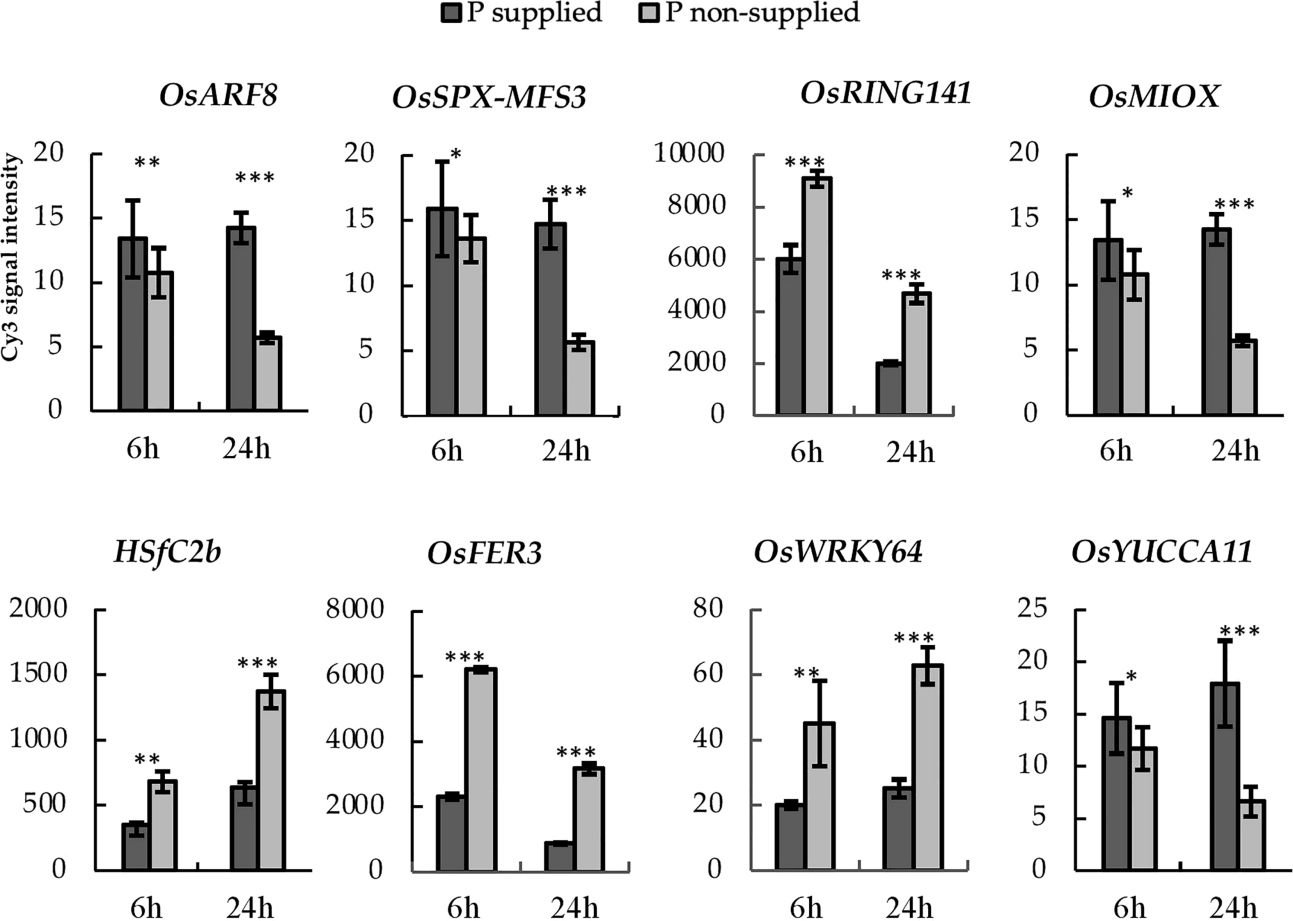
PUE-MQTL candidate genes showing significant responses to P application at 6- and 24-hours post-treatment. Asterisks denote significance using two-tailed t-test, *P<0.10; **P<0.05, ***P<0.01.

We performed haplotype analysis on the selected PUE CGs and identified potential donors for pyramiding the beneficial PUE CG alleles. We found 25 genes with at least a 2-fold change in expression levels at 24h post-treatment (**Table S6**). Among these, the eight genes (*OsARF8, OsSPX MFS3, OsRING141, OsMIOX, HsfC2b, OsFER2, OsWRKY64*, and *OsYUCCA11*) (**Figure 7**) had at least two haplotypes in the 3K RGP (**Table 5**) and were therefore used for further analysis. The number of synonymous SNPs ranged from three to nine (**Table S7**). *OsARF8*, *OsSPX-MFS3*, and *HSfC2b* had five SNPs, while *OsYUCCA11* had the most with nine SNPs. *OsRING141*, *OsMIOX*, *OsFER2, OsWRKY64* had four SNPs. The number of haplotypes ranged from two to six (**Table S8**). Four CGs had four haplotypes each (*OsARF8*, *OsMIOX*, *HSfC2b*, *OsFER2*, and *OsWRKY64*). Two CGs had six haplotypes each (*OsSPX-MF3* and *OsYUCCA11*). *OsRING141* had only two haplotypes.

**Table 5:** PUE-related candidate genes used for the haplotype analysis in the rice 3K genome panel.

MSU ID	RAP ID	MQTL	Description/function	Gene Symbol	Reference
*LOC_Os02g41800*	*Os02g0628600*	*MQTL2.7*	Similar to auxin response factor 8	*OsARF8*	–
*LOC_Os06g03860*	*Os06g0129400*	*MQTL6.1*	Splicing variant of SPX-MFS protein 3, vacuolar phosphate efflux transporter, Pi homeostasis	*OsSPX-MFS3*	Wang et al., 2015
*LOC_Os06g16060*	*Os06g0271600*	*MQTL6.5*	RING-type E3 ubiquitin ligase 141; Zinc finger, PHD-type domain containing protein	*OsRING141*	Park et al., 2019
*LOC_Os06g36560*	*Os06g0561000*	*MQTL6.7*	Myo-inositol oxygenase, drought stress tolerance	*OsMIOX*	Duan et al., 2012
*LOC_Os06g35960*	*Os06g0553100*	*MQTL6.7*	Similar to heat stress transcription factor C-2b.	*HSfC2b*	Xiang et al., 2013
*LOC_Os12g01530*	*Os12g0106000*	*MQTL12.1*	Ferritin 2, iron storage protein, iron homeostasis	*OsFER2*	Paul et al., 2012
*LOC_Os12g02450*	*Os12g0116700*	*MQTL12.1*	Similar to *WRKY* transcription factor 64, response to the rice pathogens; iron stress tolerance	*OsWRKY64*	Viana et al., 2017
*LOC_Os12g08780*	*Os12g0189500*	*MQTL12.2*	Flavin-containing monooxygenase, auxin biosynthesis, Endosperm development, regulation of grain filling	*OsYUCCA11*	Xu et al., 2021

**Figure 8:**
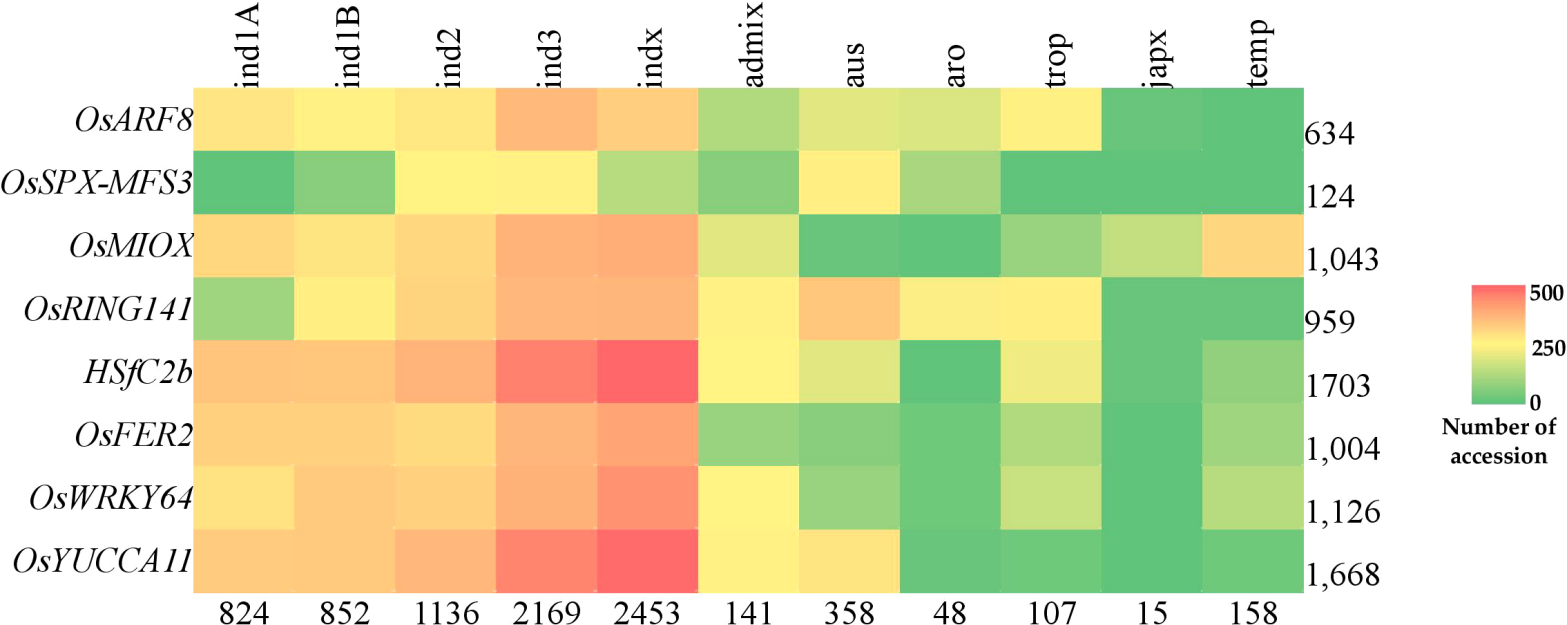
Abundance of superior PUE CG haplotypes in the rice 3K rice genome panel. Values indicate the number of accessions.

We inferred the superior haplotypes from the beneficial allele donors in the original QTLs used in MQTL analysis. Kasalath and IR20, which were donors of the beneficial alleles in MQTLs from which the eight PUE CGs were located, had haplotype information in the 3K RGP. Thus, we used them to infer superior haplotypes. The Kasalath-types were regarded as the superior haplotype for *OsARF8* (Haplotype 2), *OsRING141* (Haplotype 4), *OsSPX-MFS3* (Haplotype 4)*, and HSfC2b* (Haplotype 2). IR20-types were selected for *OsMIOX (Haplotype 1) OsFER2 (Haplotype 2), OsWRKY64 (Haplotype 2)*, and *OsYUCCA11 (Haplotype 2)* (**Table S8**). The abundance of the superior haplotypes was evaluated in the 3K RGP (**Table S9**; **Figure 8**). Superior haplotypes were found most abundant in the *indica* variety group. The frequency of superior alleles in *japonica* was considerably lower than that of the other subpopulations; in fact, superior haplotype for *OsSPX-MFS3* was completely absent. We identified rice accessions from the 3K RGP that cover the beneficial haplotypes for the eight CGs (**Table 6**). Four accessions were identified as the potential donors for the *indica* breeding programs, whereas two and three accessions were selected for the PUE breeding programs for *aus* and *japonica*, respectively. The population development using only Kasalath and IR20 can be suggested to pyramid the superior haplotypes of the eight PUE CGs.

**Table 6:** Suggested donors based on a haplotype-based selection for the pyramiding of favorable PUE CG alleles.

Subspecies or varietal group	Line	*OsARF8*	*OsSPX-MFS3*	*OsRING141*	*OsMIOX*	*HSfC2b*	*OsFER2*	*OsWRKY64*	*OsYUCCA11*
Superior haplotype controls	IR20	+		+			+	+	+
	Kasalath	*+*	*+*		*+*	*+*	*+*		
*indica*	IRGC 132300	+	+	+	+	+	+	+	+
	IRGC 132300	+	+	+	+	+	+	+	+
	IRIS 313-9415	+	+	+	+	+	+	+	+
	IRGC 126983	+	+	+	+	+	+	+	+
*aus*	IRGC 127181	+	+		+	+			+
	IRGC 135811			+	+		+	+	
*japonica*	IRGC 128463	+			+	+			
	IRGC 135811			+	+	+		+	+
	IRGC 121959						+	+	+

‘+’ represents the presence of superior haplotype of each gene **Figure 1**. Phenotypic trait classes and chromosome-wise distribution of QTLs utilized in the MQTL analysis for PUE in rice.

## 4 Discussion

The development of rice varieties requiring less agricultural inputs is essential in the face of a changing climate and the rising cost of agricultural inputs such as fertilizers. Unlike N fertilizers, P fertilizer sources are estimated to last for only another four to five generations (Shimizu et al., 2004; Van Kauwenbergh, 2010). This is further complicated by the lack of plant-available P, despite fertilizer application due to the inherently low PUE in rice (Rose et al., 2011). It is therefore vital to utilize genetic variation in rice to address these issues. PUE-related QTLs and genes have been previously studied and mapped. However, their practical breeding utility has been hampered by their unstable effect across environments, genetic backgrounds, QTL to QTL or gene to gene interactions, and linkage drag caused by the large introgression size of QTLs that have not undergone fine-mapping. One example is the instability of the Kasalath-derived major PUE QTL *Pup1*, which confers tolerance to low P application as well as to mild drought. *Pup1* improves yield under low P in several *indica* varieties such as IR64, IR74, Situ Bagendit, Batur, and Dodokan under tropical conditions (Chin et al., 2011). However, *Pup1* introgression into Dasanbyeo, a Tongil-type *indica*, neither improved phosphorus uptake nor yield under low P application in temperate regions (Navea et al., 2022). In addition, a study has revealed the inability of *Pup1* to confer tolerance to low P in early shoot growth when introgressed simultaneously with the submergence tolerance major QTL *Sub1* into IR64 (Shin et al., 2021). The inconsistencies in QTL effects necessitate the identification of fine-mapped genomic regions related to PUE that are effective across diverse genetic backgrounds and environments.

We conducted an MQTL analysis on 192 PUE QTLs identified in 16 independent studies, in 15 non-overlapping bi-parental mapping populations. MQTL analysis offers the benefit of identifying reliable and robust genomic regions with reduced numbers of QTLs and narrower introgression sizes (Goffinet and Gerber, 2000; Kumari et al., 2021; Aloryi et al., 2022; Anilkumar et al., 2022; Joshi et al., 2023). To the best of our knowledge, our study is the first attempt to identify MQTLs underlying PUE in rice. Initially, we identified 60 PUE MQTLs and further selected 38 MQTLs that were supported by at least two independent studies and had PVE values of at least 5%. The majority of QTLs underlying these MQTLs had small PVE values ranging from 3% to 6%, implying that PUE in rice is controlled by many minor loci. This observation is consistent with results from several PUE QTL mapping studies (Navea et al., 2017; Wang et al., 2014). The distribution of the initial QTLs largely varied throughout the whole chromosome (**Figure 1**). However, it is not largely attributable to the SNP density (**Figure S2**). This is in contrast to the results from other MQTL studies in rice (Khahani et al., 2021; Selamat and Nadarajah, 2021; Aloryi et al., 2022; Joshi et al., 2023), wherein the uneven distribution of the initial QTLs mostly depended on the number of markers used in constructing the consensus map. Chromosome 6 was the chromosome with the largest number of initial PUE QTLs, as reported in the various previous PUE studies in rice (Ismail et al., 2007; Wissuwa et al., 2015; Heuer et al., 2017).

Linkage drag, due to the large CI of the QTLs utilized in breeding programs, is one of the hindrances in achieving successful in marker-assisted breeding (MAB) and genomic selection (GS) programs (Collard and Mackill, 2008; Kumar et al., 2020). Here, the average CI of the PUE MQTLs was reduced compared to their initial QTL members (**Figure 4**), except in case of chromosomes 4 and 12. MQTL analysis was not able to narrow down the CI on chromosomes 4 and 12 partly due to the small number of the initial QTL members as well as the already small CI of the QTLs. With the exceptions of the MQTLs detected on chromosomes 4 and 12, the average CI was reduced from 14.04 cM to 2.70 cM, suggesting the power of MQTL analysis in identifying precise genomic segments modulating a trait of interest. Our results are consistent with the observations in previous MQTL studies on various traits in cereal crops (Chen et al., 2017; Venske et al., 2019; Soriano et al., 2021; Miao et al., 2022).

The successful integration of pleiotropic QTLs into breeding programs has been documented in rice (Ookawa et al., 2010; Yan et al., 2011; Vishnukiran et al., 2020). In this study, we categorized QTLs identified under field conditions, based on traits well-distributed throughout the rice growth stages. These included seedling stage (RT, RSR, RRP), vegetative stage (SDW, BM, IPT), and reproductive stage traits (SPC and YLD). This enabled us to identify PUE MQTLs that may have pleiotropic effects, as observed in MQTL1.5, MQTL1.6, and MQTL1.7, on multiple traits including BM, SCP, RSR, IPT, YLD, and RRP. This can aid in a more efficient improvement of PUE in rice through the accumulation of beneficial PUE MQTL alleles. Among the QTLs used in this study, genomic regions regulating root traits under P deficient conditions were the least abundant across rice chromosomes, which can be attributed to the challenges in establishing reliable and high-throughput root phenotyping techniques under field conditions (Heuer et al., 2017), as well as the lack of genetic diversity (Ismail et al., 2007). It is worth noting that the number of PUE CGs underlying MQTL2.7, MQTL4.3, and MQTL11.4 are relatively large despite their narrow CI. This is unlike the observations in previous MQTL studies in rice, wherein the number of CGs underlying an MQTL had a strong positive correlation with the size of the CI (Daware et al., 2017; Islam et al., 2019; Khahani et al., 2021; Selamat and Nadarajah, 2021; Aloryi et al., 2022; Anilkumar et al., 2022; Joshi et al., 2023). These MQTLs have the potential to be effective genomic targets for use in the MAB after being validated in a wide range of genetic backgrounds and environments by utilizing the aforementioned linked flanking markers (**Table 3**).

We identified CGs underlying the PUE MQTLs using GO terms directly related to PUE traits, as well as for the secondary PUE traits. We further selected genes that could be validated through the root expression data under control and P deficient/non-supplied conditions. Most of the PUE CGs (83%) were upregulated (**Figure 6F**), implying that genes underlying the PUE MQTLs confer an active response to nutrient deficiency, rather than conservation of resources. This agrees with the pattern of gene regulation under nutrient deficiency in previous studies in various plants (López-Bucio et al., 2003; Chen et al., 2022; Wang et al., 2019). The results of our GO analysis give an insight into the complexity of PUE in rice. CGs underlying PUE MQTLs were heavily enriched with genes involved in amino acid transmembrane transport, organic acid transport, and response to auxin. Amino acid transporters in rice, although not reported to be directly involved in PUE, have been associated with the regulation of flowering time and defense against abiotic stresses and pathogen attack (Guo et al., 2021). In particular, the genes involved in the amino acid transport pathways function in various plant species defending against both biotic and abiotic stresses through the regulation of the salicylic acid pathway (Kan et al., 2017), including drought, salinity, UV radiation, heavy metals, and pathogens (Szabados and Savouré, 2010). In plants, organic acid transporters are upregulated by Al and/or P deficiency (Yu et al., 2016). This is caused by enhanced phosphorylation levels of the plasma membrane H+-ATPase, which creates an electrochemical potential across the cell membrane. This leads to an increase in the activity of the organic acid transporters and the passive release of organic anions from root tips. Ultimately, this process causes organic acids to exude from the roots and form a stable complex with Al, which allows P to become soluble for plant assimilation (Ligaba et al., 2004). Plant root architecture undergoes adaptive changes, including the inhibition of primary root growth and the increase in the number and length of lateral roots (Péret et al., 2011) modulated by the change in sensitivity of auxin receptors such as *TIR1*, under a P deficient condition (Wu et al., 2023). Upregulation of auxin receptors degrades auxin repressors, releasing auxin response factor, *ARF19*, which then leads to the activation of genes related to lateral root morphogenesis (Pérez-Torres et al., 2008).

We further narrowed down the PUE CGs to eight genes and investigated their natural variation in a diverse set of rice (3K RGP). These genes included *OsARF8*, *OsSPX-MFS3*, *OsRING141*, *OsMIOX*, *HsfC2b*, *OsFER2*, *OsWRKY64*, and *OsYUCCA11* (**Figure 7**). *OsSPX-MFS3* is a member of the rice SPX-MFS family which mediates Pi transport between the cytosol and vacuole (Yang et al., 2017). *OsSPX-MFS3* plays a major role in the transport of Pi from the cytosol to vacuole (Guo et al., 2023) and is downregulated under P deficiency. The regulation pattern of *OsSPX-MFS3* is consistent with the observation in the microarray data utilized in this study. It is worth noting that, except for *OsSPX-MFS3*, the rest of the CGs did not have GO terms directly related to PUE but were nevertheless associated to secondary PUE traits and other abiotic stresses. Auxin is one of the phytohormones that regulate root architecture modifications in response to Pi deficiency (Nacry et al., 2005). In this study, we identified two CGs, namely *OsARF8* and *OsYUCCA11*, implicated in auxin response and which were significantly downregulated under P non-supplied condition. *OsARF8* is a member of the ARF family implicated in the crosstalk between auxin signaling and P status (Wang et al., 2014).

Genes under the zinc-finger family may play an important role in the regulation tolerance to multiple stresses in rice (Deng et al., 2018). The present study identified a P-deficiency-upregulated zinc-finger gene, *OsRING141*, among the genes subtending MQTL6.5. Similarly, previous studies found two C2H2- type zinc finger protein genes, ZOS3-12 and ZOS5-08, to be responsive to P and N deficiencies (Huang et al., 2012). MQTL12.1 harbors two genes modulating iron (Fe) homeostasis (*OsFER2*) and iron stress tolerance (*OsWRKY64*). Both genes were significantly upregulated in the P non-supplied condition. Previous studies have shown that the reduction of Fe concentration can lead to the recovery of primary root elongation under low P conditions, suggesting that Fe may play a role in the Pi deficiency-induced reduction of primary root growth (PRG) (Ward et al., 2008). Moreover, research on Arabidopsis has demonstrated that when Fe is deposited in the root tip meristem, it can trigger the accumulation of reactive oxygen species (ROS) and callose, likely through LPR1-dependent redox signaling (Müller et al., 2015). The accumulation of ROS and callose can interfere with cell-to-cell communication that is essential for maintaining the stem cell niche and inhibiting PRG (Müller et al., 2015).

MQTL6.7 harbors two abiotic stress-related genes, namely *OsMIOX* (drought) and *HSfC2b* (heat). A previous study reported that the overexpression of *OsMIOX* in transgenic rice greatly improved growth performance under drought conditions by decreasing oxidative damage (Duan et al., 2012). Severe phosphorus deficiency can lead to changes in the photosynthetic apparatus, such as decreased rates of carbon dioxide assimilation, reduced expression of photosynthesis-related genes, and photoinhibition at the photosystem II level. These changes can potentially cause photo-oxidative stress, which can damage the plant’s cells. By preventing oxidative damage, the plant may be able to better tolerate the effects of phosphorus deficiency on its photosynthetic apparatus (Hernández and Munné-Bosch, 2015). Therefore, as *OsMIOX* may prevent oxidative damage, it might be beneficial to phosphorus-starved plants. A heat shock factor (HSF) member, *HSfC2b*, harbored within MQTL6.7, was upregulated under P deficient condition. The first attempt to elucidate the genome wide expression of these HSFs was conducted by Chauhan et al. (2011). HSF encoding genes showed significant upregulation in abiotic stresses such as cold, drought, and salinity. Our study is the first to show the link between an HSF gene and PUE in rice. In Arabidopsis, the overexpression of HSFs in transgenic plants confer simultaneous tolerances to multiple abiotic stresses such as heat and anoxia (Banti et al., 2010). Nevertheless, further study is needed to elucidate the relationship between HSF and PUE in rice.

The use of NGS-based genotyping methods have aided in the identification of SNPs associated with agronomic traits in rice. However, the applicability of SNPs in breeding programs is constrained by their bi-allelic nature by cross breeding, the presence of uncommon alleles, and the abundance of linkage drag (Annicchiarico et al., 2017). Considering the gene haplotypes for genome-wide analysis will help overcome SNP marker’s limitation. Haplotypes are specific combinations of jointly inherited nucleotides or DNA markers from polymorphic sites in the same chromosomal segment (Stephens et al., 2001; Lu et al., 2010). Haplotype-based breeding holds a promise in accumulating beneficial alleles for a trait of interest in living organisms. This can be achieved through the identification of haplotypes for different genes and utilizing them through techniques such as allele mining, pyramiding, or GS. This approach has resulted in genetic gains in crops (Abbai et al., 2019; Anandan et al., 2022; Du et al., 2022). We identified subspecific-wise potential donors of the superior haplotypes for PUE CGs: *OsARF8*, *OsSPX*-*MFS3*, *OsRING141*, *OsMIOX*, *HsfC2b*, *OsFER2*, *OsWRKY64*, and *OsYUCCA11* (**Table 6**). We inferred the superior haplotypes for these genes based on the haplotype of beneficial allele donors (Kasalath and IR20) in the initial QTL studies used for the meta-QTL analysis. The frequencies of superior haplotypes differed among sub-populations, with particular groups exhibiting a higher prevalence (**Figure 8**; **Table S9**). Similarly, previous studies have suggested that the distribution of haplotypes is influenced by evolutionary and population genetic factors, such as rates of mutation and recombination, as well as selection pressures (Magwa et al., 2016; Sinha et al., 2020). Superior haplotypes for almost all the CGs were predominant in *indica* subspecies. The frequency of the superior haplotypes in *japonica* was considerably smaller, compared to that of other subspecies. As mentioned, *OsSPX-MFS3* was completely absent in *japonica* varieties in 3K RGP. This implies a big opportunity to improve PUE in the *japonica* varieties using the donors identified in this study. Our results suggest that *indica* varieties are a rich source of PUE CG superior haplotypes and could be utilized in the genomics-assisted breeding programs for PUE in rice. In the case of the *japonica*, it is necessary to generate pre-breeding lines that encompass the superior PUE CG haplotypes.

We identified potential CGs associated with PUE as well as superior haplotypes that could be used to accumulate beneficial alleles to improve PUE in rice. However, it should be noted that the PUE CG mining pipeline used in the present study was limited by the availability of P-supplied and P-non-supplied root transcriptome data. Initially, we identified 273 PUE genes (**Table S1**), but we could only analyze 238 CGs (**Table S2**) that had gene expression data in the RiceXPro database. Therefore, a complete set of transcriptome data would provide offer higher precision in the identification of PUE CGs in rice. Another limitation of our pipeline is the limited scope of the reference genome used in both the annotation of genes and the root expression analysis. We utilized gene annotation from the IRGSP v. 1.0 (Nipponbare) reference genome, a widely used reference genome for rice research, to identify PUE CGs. Here, it was not possible for us to identify genes that were absent in the reference genome and therefore were possibly neglected in our analysis. For instance, the P uptake gene *OsPSTOL1* harbored in the P uptake major QTL *Pup1* is absent in the reference genome Nipponbare, as well as in the genomes of several other commonly used rice varieties (Chin et al., 2011; Gamuyao et al., 2012). Similarly, *Sub1A*, a gene conferring submergence tolerance in rice, was not found in the fully sequenced genome of Nipponbare (Xu et al., 2006). The limited availability of gene information from the reference genome may fail to precisely capture the genetic variability linked to PUE. Consequently, it is important to generate supplementary reference genomes, or a “rice meta-genome,” that encompass diverse rice varieties and species to address this limitation. Nevertheless, our approach provides breeders with valuable information regarding the selection of optimal donors for their desired traits from the gene bank. However, the assumed genetic gains resulting from the accumulation of superior haplotypes of PUE CGs require validation in practical breeding programs.

## 5 Conclusion

We identified 38 meta-QTLs (MQTLs) for phosphorus use efficiency (PUE) that were supported by multiple QTLs from independent studies, which had a phenotypic variation explained (PVE) value of at least 5%. We subjected the 38 PUE MQTLs to candidate gene (CG) mining. The genomic regions associated with PUE MQTLs were found to be enriched with genes involved in the transmembrane transport of amino acids and organic acids, as well as genes involved in the response to auxin. Some superior haplotypes containing eight CGs for PUE could be considered for the genomics-assisted breeding in rice.

## Data availability statement

The original contributions presented in the study are included in the article/**Supplementary Material**. Further inquiries can be directed to the corresponding author.

## Author contributions

IN and JC conceptualized and designed the manuscript. JC and WZ supervised the study. IN and PM curated data and performed analysis. IN wrote the manuscript. JC, WZ, PM, N-HS, J-HH, SY, and WJ reviewed end edited the manuscript. All authors contributed to the article and approved the submitted version.
