# Supplementary material for: A meta-QTL analysis highlights genomic hotspots associated with phosphorus use efficiency in rice (Oryza sativa L.)
Source: Front Plant Sci. 2023 Aug 18;14:1226297. doi: 10.3389/fpls.2023.1226297 (PMC10471825; doi:10.3389/fpls.2023.1226297)
Supplement: Supplementary file 1 [file DataSheet_1.docx]

Supplementary Material

A meta-QTL analysis highlights genomic hotspots associated with phosphorus use efficiency in rice (*Oryza sativa* L.)

Ian Paul Navea, Phyu Phyu Maung, Shiyi Yang, Jae-Hyuk Han, Wen Jing, Na-Hyun Shin, Wenhua Zhang, Joong Hyoun Chin^*^

*** Correspondence:** Joong Hyoun Chin: jhchin@sejong.ac.kr

##
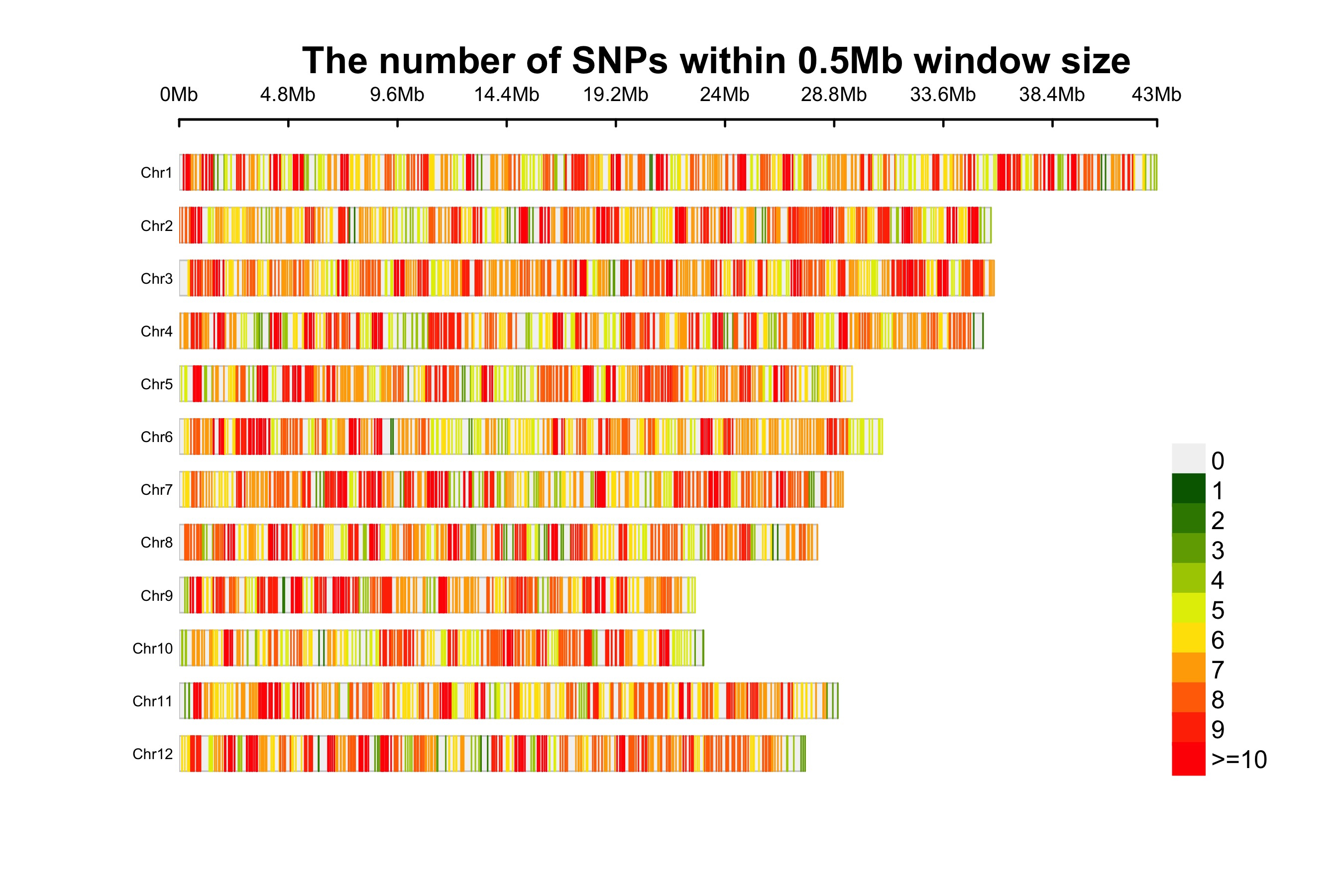
Supplementary Figures

**Figure S1.** Contribution and the density of SNPs on the consensus map used to project the MQTLs for PUE. Color intensity denotes the number of SNPs within 0.5Mb window size.


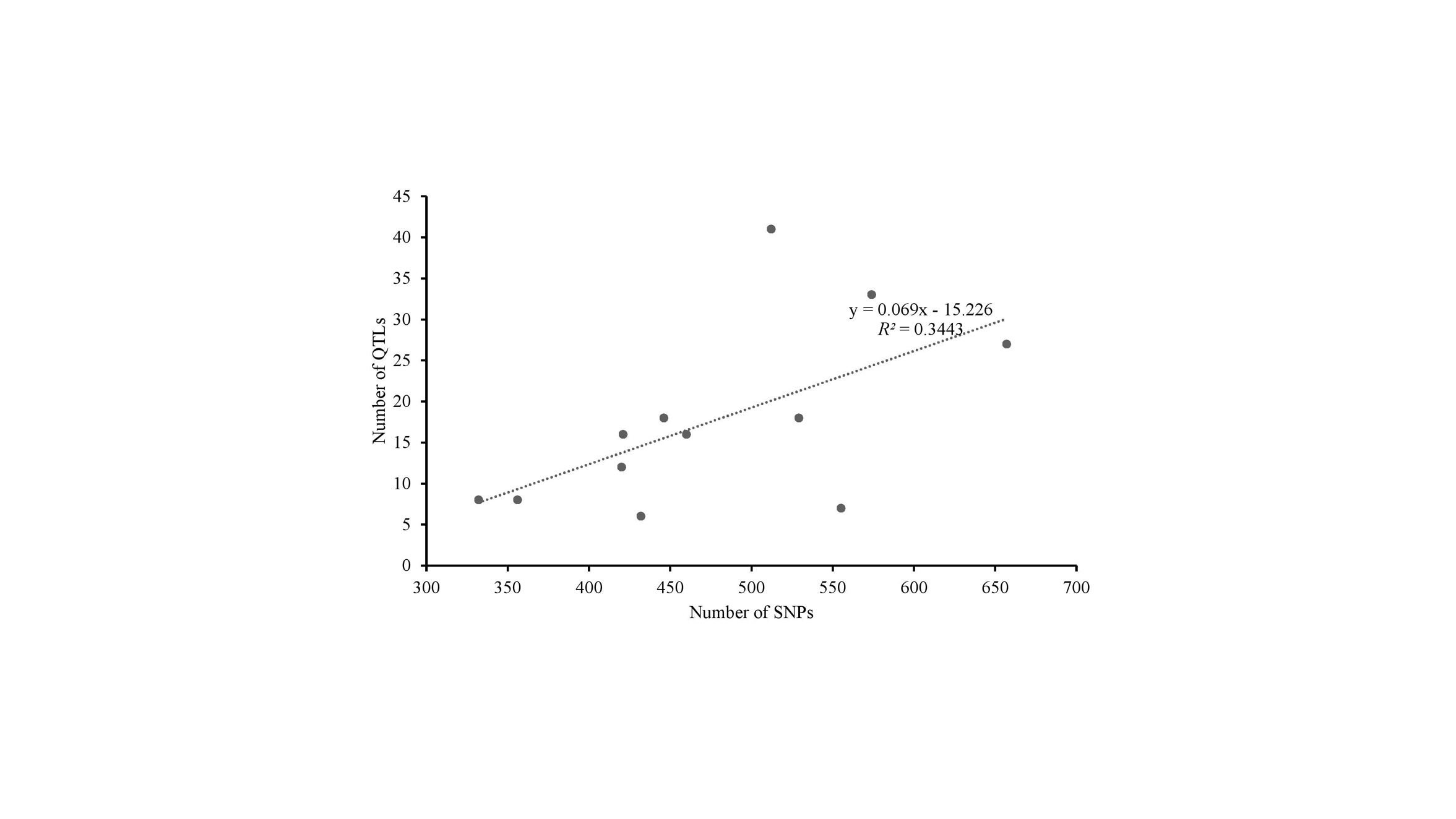


**Figure S2.** Scatterplot showing the relationship between the number of SNPs and the number of QTLs. The trendline formula for this data is y = 0.069x - 15.226, indicating a positive linear relationship between number of SNPs and QTLs. The correlation coefficient (*R^2^*) is 0.3443, indicating a weak positive correlation between the variables.

**
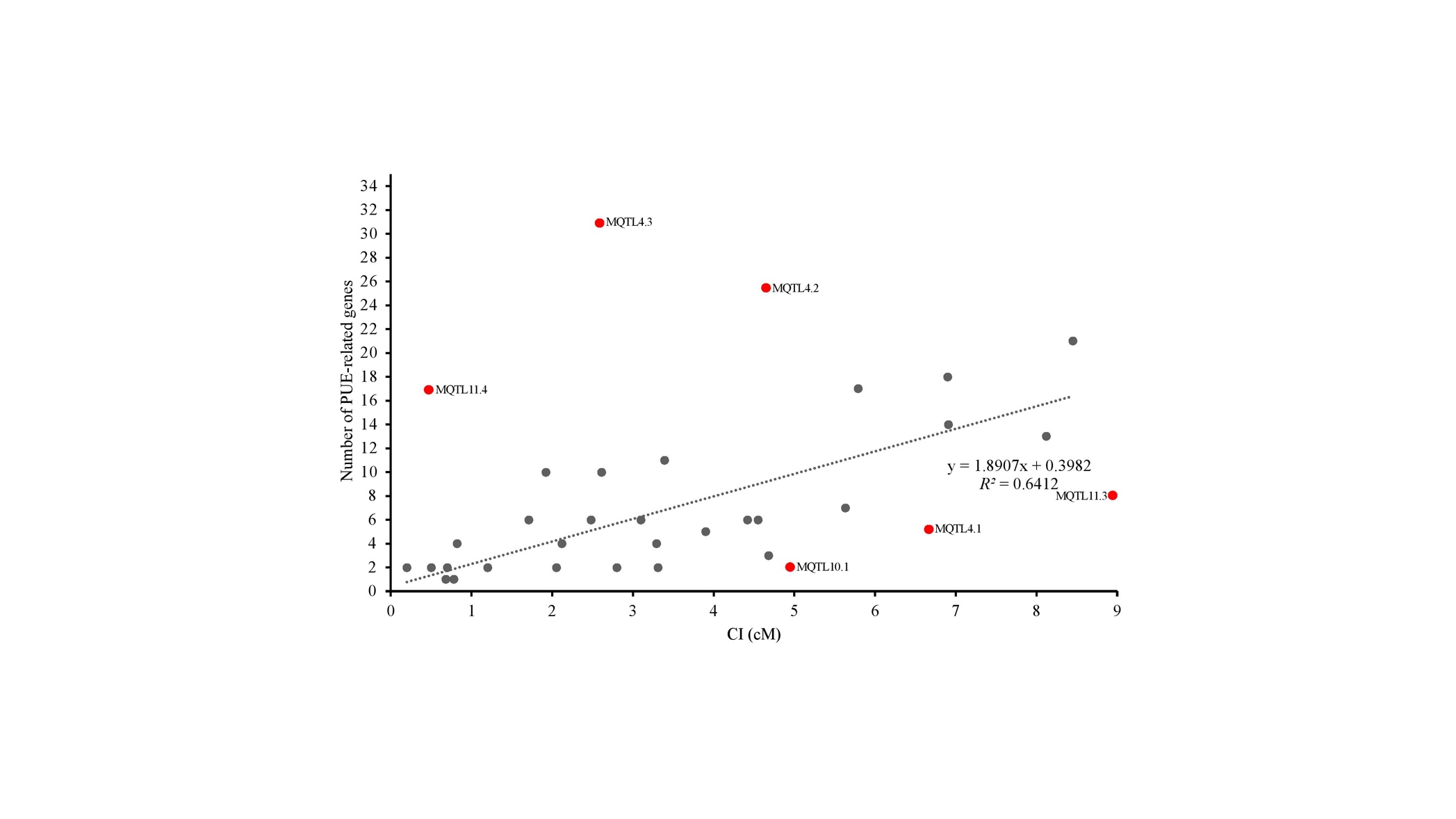
**

**Figure S3.** Scatterplot showing the relationship between the confidence interval (CI) and the number of PUE genes. The trendline formula for this data is y = 1.8907x + 0.3982, indicating a positive linear relationship between CI and genes. The correlation coefficient (*R^2^*) is 0.6412, indicating a strong positive correlation between the variables.
